# Neuronal substrates of egg-laying behaviour at the abdominal ganglion of *Drosophila melanogaster*

**DOI:** 10.1038/s41598-023-48109-1

**Published:** 2023-12-11

**Authors:** Cristina Oliveira-Ferreira, Miguel Gaspar, Maria Luísa Vasconcelos

**Affiliations:** Neuroscience Programme, Champalimaud Foundation, Lisbon, Portugal

**Keywords:** Behavioural genetics, Motor control, Neural circuits

## Abstract

Egg-laying in *Drosophila* is the product of post-mating physiological and behavioural changes that culminate in a stereotyped sequence of actions. Egg-laying harbours a great potential as a paradigm to uncover how the appropriate motor circuits are organized and activated to generate behaviour. To study this programme, we first describe the different phases of the egg-laying programme and the specific actions associated with each phase. Using a combination of neuronal activation and silencing experiments, we identify neurons (OvAbg) in the abdominal ganglion as key players in egg-laying. To generate and functionally characterise subsets of OvAbg, we used an intersectional approach with neurotransmitter specific lines—*VGlut*, *Cha* and *Gad1*. We show that OvAbg/VGlut neurons promote initiation of egg deposition in a mating status dependent way. OvAbg/Cha neurons are required in exploration and egg deposition phases, though activation leads specifically to egg expulsion. Experiments with the OvAbg/Gad1 neurons show they participate in egg deposition. We further show a functional connection of OvAbg neurons with brain neurons. This study provides insight into the organization of neuronal circuits underlying complex motor behaviour.

## Introduction

Motor execution of behavioural programmes must be tightly controlled so that the appropriate sequences of actions are executed once the internal state and environmental cues are considered. Egg-laying is executed by female fruit flies up to 80 times per day^1^. For each egg laid, the female follows an egg-laying motor programme described as comprising a search-like period followed by egg deposition and subsequent clean and rest^2^. Presumably, during the search period the fly evaluates the environment in order to find the best site to deposit the egg. For flies and the other oviparous animals, when and where an egg is deposited has a profound impact on the survival of the offspring. A female decides an egg-laying site based on substrate texture^3,4^, food availability^2,5,6^, protection from climate elements^7,8^, predator avoidance^9–16^, and disease avoidance^17–20^. The female will also evaluate information from other flies^21–25^.

In recent years a number of mechanosensory^3,4,26^, chemical^2,5–7,9–13,17,18,21^ and visual^8,14–16,23^ cues that modulate egg-laying site selection have been identified, creating a broad view of the external cues guiding egg-laying decisions. Internal cues prompt the search period in the female^27^. Movement of the egg through the reproductive system is in part controlled by octopamine, which contracts the ovaries and relaxes the oviduct, and glutamate, which contracts the oviduct^28–30^. Sensory neurons expressing the mechanosensory channel *Piezo* at the oviduct detect contraction/distention leading to a search for an egg-laying site^27^. While great progress has been made in understanding site selection and its neuronal underpinnings, very little is known regarding the consummatory component of egg-laying: egg deposition.

Here we addressed the neuronal basis of the egg deposition motor programme, one of the phases of egg-laying, at the lower level of motor control. We began with a description of the behavioural elements of the full egg-laying motor programme in wild type flies. Next, we searched lines that label neurons in the abdominal ganglion of the ventral nerve cord, where potentially brain commands and reproductive system inputs are integrated to perform egg deposition. Activation and silencing experiments revealed a line labelling neurons that are crucial for egg deposition behaviour. We used an intersectional approach where we genetically intersect OvAbg neurons with three neurotransmitter driver lines: *Cha* (*ChaT,* acetylcholine biosynthesis), *VGlut* (vesicular glutamate transporter), and *Gad1* (GABA biosynthesis). W e showed that OvAbg/Cha neurons participate in the exploration and egg deposition phases, although when activated led to egg pushing and expulsion. OvAbg/VGlut neurons contribute for the initial elements of egg deposition. OvAbg/Gad1 neurons also participate in egg deposition, but prolonged activation of this subset supresses egg-laying. We showed that activity of OvAbg neurons is required for the execution of oviDNs command. This study shows for the first time the link between the activity of abdominal ganglion neurons and specific behavioural motor elements. This knowledge provides insight into the architecture of the egg deposition circuitry at the level of the lower motor control.

## Results

### Characterization of egg-laying behaviour

**Figure 1:**
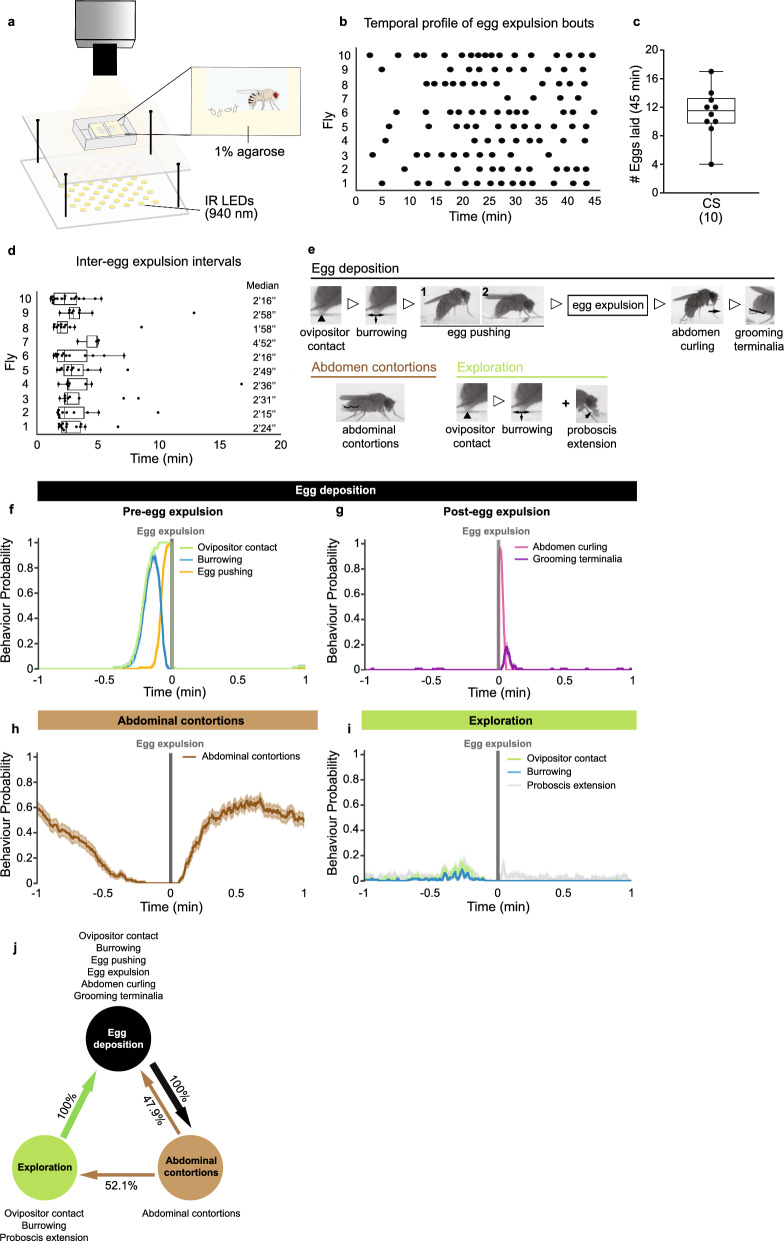
Egg-laying behaviour in wild type flies. (**a**) Schematic of the setup used to record egg-laying behaviour. Each arena is composed of two chambers allowing the recording of two flies simultaneously. The egg-laying substrate used in this study was 1% agarose. Infrared (IR) LEDs were used for illumination and IR cameras were used to record fly behaviour. (**b**) Raster plot showing the temporal profile of egg expulsion bouts during a 45-min period. Each black dot marks the moment of egg expulsion. (**c**) Number of eggs laid by Canton S (CS) flies. Median = 11.5. Box plots indicate median (middle line), 25th, 75th percentile (box) and minimum and maximum values (whiskers). (**d**) Time intervals between egg expulsion bouts for each fly. Each dot represents the interval between consecutive egg deposition bouts. Median values of the distribution for each fly are shown on the right side. (**e**) Frame snapshots illustrating the egg-laying motor patterns analysed within each egg-laying phase. Open triangles between snapshots denote sequential behaviours. Note that egg pushing behaviour can be displayed in two different postures: (1) erect body posture (fly head is elevated in relation to the abdomen, or (2) leaning body posture (fly leans towards the substrate). (**f**–**i**) Probabilities of indicated egg-laying behaviours during a 1-min time window around egg expulsion. Time = 0 min marks the moment of egg expulsion (represented by the grey vertical line). Shaded area represents the standard error of the mean (sem). n = 112 egg expulsions. (**j**) Transition probabilities of the egg-laying phases: egg deposition (black), exploration (green) and abdominal contortions (brown). Arrow thickness represents the degree of transition likelihood. n = 10 flies in (**b**–**d**) and (**f**–**j**); n = 112 egg expulsions in (**c**,**d**) and (**f**–**i**).

In order to understand how the execution of egg-laying motor programme is coordinated, we analysed wild type egg-laying behaviour in detail. Figure 1a shows a schematic representation of the behavioural setup. Single females were placed in an arena lined with 1% agarose on three walls. Videos were recorded for 45 min. We first analysed the temporal structure of egg expulsion (Fig. 1b). For the duration of the video, each female laid from 4 to 17 eggs, with a median of 11.5 (Fig. 1c). All eggs were laid on the agarose walls and 94% of eggs were buried in the agarose. The median inter-egg expulsion interval calculated for each female ranges from 2 to 3 min with the exception of one fly (fly#7) that showed a median of 5 min (Fig. 1d). These data indicate a low inter-individual variability when rearing conditions and environment are controlled.

To obtain a detailed description of egg-laying behaviour, we analysed egg-laying motor elements which we associated with different egg-laying phases. Egg-laying behaviour involves (1) an ‘exploration phase’ where the fly presumably searches for appropriate egg-laying sites, (2) the ‘egg deposition phase’ in which a motor programme is activated to lay an egg, and (3) a ‘rest phase’. We chose to call the rest phase ‘abdominal contortions’ because we and others^26,31^ observed that this phase is accompanied by strong and prolonged abdominal contortions, as described below. Figure 1e shows videoframes capturing each of the different egg-laying motor elements. Some behavioural denominations were based on other descriptions of egg-laying^26,31^. ‘Ovipositor contact’ is defined by abdomen bending accompanied by extrusion of the ovipositor and contact with underlying substrate. It can culminate in egg expulsion or not. ‘Burrowing’ is characterized by scratching the surface eventually leading to digging the substrate with the ovipositor. ‘Egg pushing’ is characterized by a rigid posture that initiates at the end of burrowing behaviour and accompanies egg expulsion. ‘Abdomen curling’ is characterized by curling the abdomen followed by walking forward, either by lifting the curled abdomen, or by dragging it through the substrate. ‘Grooming terminalia’ is self-explanatory. ‘Abdominal contortions’ are undulated abdominal movements accompanied by extrusion of the ovipositor. ‘Proboscis extension’ is characterized by the proboscis being extended to contact the substrate. Egg deposition motor elements all progress in a sequence culminating in egg expulsion and ending with grooming of the terminalia (Fig. 1e–g). We defined exploration motor elements as elements where females probe the substrate either with the ovipositor or the proboscis without progressing in a continuous sequence to egg expulsion (Fig. 1e and i).

To understand the temporal sequence of the different behavioural elements, we plotted the probability of each motor element around the moment of egg expulsion (Fig. 1f–i). During the egg deposition phase, the motor elements leading to egg expulsion are performed every time an egg is deposited (Fig. 1f). After an egg is expelled, the female curls the abdomen while walking away, which can be followed by grooming terminalia (Fig. 1g). While ovipositor contact, burrowing, egg pushing and abdomen curling are fixed elements of egg deposition, grooming terminalia is optional. A few seconds after the egg is expelled, the probability of abdominal contortions rises (Fig. 1h). The timing of abdominal contortions is variable, though abdominal contortions generally increase a few seconds after egg expulsion and decrease in the minute leading up to egg expulsion. It was recently shown that abdominal contortions correspond to the moment of ovulation, since the next phase, exploration, is activated by ovulation^27^. As abdominal contortions subside, the exploration motor programme initiates (Fig. 1i). Females contact the substrate by extending the proboscis and by bending the abdomen to reach the surface with the extruded ovipositor. A small peak of proboscis extension is also observed after egg expulsion indicating there may also be an association of this behaviour with the post-egg expulsion motor sequence. Part of these ovipositor contacts are accompanied by burrowing. These two behavioural elements are the same as those observed in the egg deposition programme, except that they are not followed by egg pushing and expulsion. An overview of exploration and egg deposition phases, together with the respective motor elements, is provided in Supplementary Movie 1. The abdominal contortions phase is represented in Supplementary Movie 2.

We next analysed the transition probabilities between the different phases (Fig. 1j). This analysis revealed that egg deposition is always followed by abdominal contortions. Abdominal contortions are either followed by exploration or egg deposition with similar likelihood. Once the female initiates exploration she does not return to abdominal contortions without depositing an egg first. Similarly, once the female deposits an egg, she does not explore without going through abdominal contortions first. In our quantification of phase transitions, we did not consider proboscis extension, as it is not exclusive to the exploration phase nor to egg-laying behaviour. Still, the results suggest that exploration is optional in this setup. Here we described seven behavioural elements and associated these elements with each phase, setting the stage to analyse the neuronal circuits of egg-laying.

### Activity of OvAbg neurons promotes egg deposition

Organs in the abdominal region of the flies connect to the central nervous system at the abdominal ganglion (Abg) of the ventral nerve cord (VNC). Since the reproductive organs are located in the abdomen and many egg-laying behavioural elements involve abdominal movement, this region is candidate to identify neurons involved in the execution of egg-laying behaviours. Therefore, we performed an activation screen of splitGal4 lines^32–34^ selected based on Abg expression. From this screen, we selected a line—we will call *OvAbg*—for further investigation. Interestingly, the *OvAbg* line is the product of an intersection with a splitGal4 version of the *doublesex* (*dsx*) driver line^34^, implying that all OvAbg neurons are *dsx* positive. *dsx* is a sex determination gene widely known to control sex specific reproductive behaviours in flies^34–38^.

**Figure 2:**
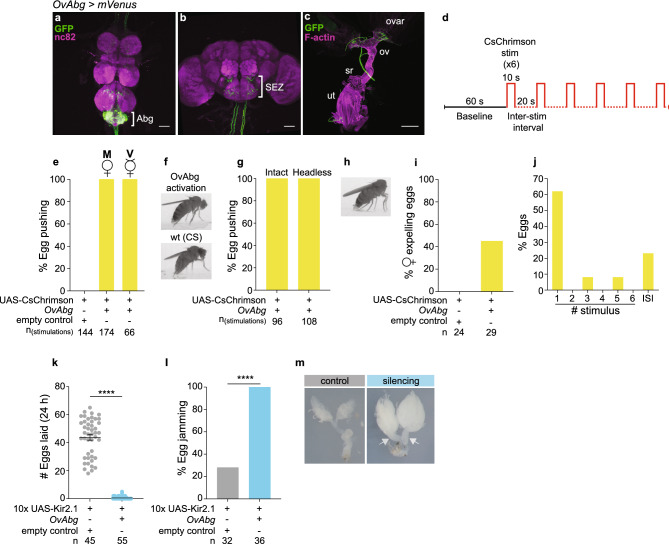
Activity of OvAbg neurons promotes egg deposition. (**a**–**c**) Confocal images of female VNC (**a**), brain (**b**) and reproductive system (**c**) of OvAbg neurons and corresponding innervations labelled with anti-GFP antibody (green) to reveal the anatomy and nc82 for neuropil. Phalloidin, which binds F-actin, was used in (**c**) to visualize the muscle fibres. Abg: abdominal ganglion; SEZ: subesophageal zone; ovar: ovary; ov: oviducts; sr: seminal receptacle; ut: uterus. Anti-GFP antibody is targeting the fluorescent protein *Venus* of *OvAbg* > *UAS-CsChrimson-mVenus* expressing flies. Scale bars, (**a**,**b**) 50 µm and (**c**) 200 µm. (**d**) Schematic of photoactivation protocol: a baseline period (1 min) is followed by 6 activations (10 s each) that are interspaced by 20-s intervals. (**e**) Percentage of stimulation events in which mated (M) and virgin (V) OvAbg flies displayed an egg pushing like posture. n = 144 (control) and 174 (OvAbg) stimulations. (**f**) Frame snapshots of an activated OvAbg female (top) displaying an egg pushing-like posture. A snapshot of a CS fly is shown (bottom) for comparison of the egg pushing posture performed by wild type flies during egg-laying. (**g**) Percentage of stimulation events and (**h**) frame snapshot in which headless OvAbg flies displayed an egg pushing-like posture. n = 96 (control) and 108 (OvAbg) stimulations. (**i**) Percentage of activated OvAbg females expelling eggs. n = 24 (control) and 29 (OvAbg) females. (**j**) Percentage of eggs expelled during stimulation periods and inter-stimulus intervals (ISI). n = 13 eggs. (**k**) Number of eggs laid in the 24 h after mating during inhibition of OvAbg neurons. n = 45 (control) and 55 (OvAbg) flies. Bars indicate mean ± sem. Mann Whitney test, ****p < 0.0001. (**l**) Percentage of females with eggs jammed in the lateral oviducts. n = 32 (control) and 36 (OvAbg) flies. Fisher’s exact test, ****p < 0.0001. (**m**) Image representing the reproductive system of a control reproductive system with no eggs in the oviducts and a silenced OvAbg female with two eggs jammed (white arrows) in the lateral oviducts. Note that, besides the egg jamming, OvAbg silencing leads to enlarged ovaries containing more mature oocytes.

The *OvAbg* line anatomy reveals a group of 153 (± 9, n = 7) neurons exclusively localized in the Abg (Fig. 2a). OvAbg neurons innervate other ganglia of the VNC (Fig. 2a), the suboesophageal zone (SEZ) (Fig. 2b), and the reproductive system, specifically in the lateral oviducts and the distal uterus (Fig. 2c). Additionally, we found OvAbg projections in the abdominal segments A6-8 of the body wall muscle (Supplementary Fig. 1a and b). To identify the polarity of OvAbg neurons, we used synaptotagmin and denmark labelling, which revealed presynaptic terminals in the SEZ and mixed labelling of processes in the Abg and lateral oviducts (Supplementary Fig. 1c–k).

To investigate the role of OvAbg neurons in egg-laying, we optogenetically activated them using CsChrimson^39^ with 6 stimuli of 10 s and a 20 s interval between stimuli (Fig. 2d). The neuronal activation always led the mated female to assume an egg pushing posture (Fig. 2e, Supplementary Movie 3). No other egg-laying motor elements were triggered by OvAbg activation. Control females, which have the same genetic background as OvAbg, but no regulatory element for splitAD^40^, never assume the egg pushing posture during light stimulation. Figure 2f depicts the egg pushing posture of an OvAbg activated female and a wild type female during unmanipulated egg-laying for comparison. To address whether mating status affects the output of OvAbg neurons, we activated virgin OvAbg females. We observed that virgins, like mated females, assume an egg pushing posture in all light ON periods (Fig. 2e). To test the contribution of the OvAbg projections in the brain to the activation phenotype, we activated headless females. We observed that similarly to intact OvAbg females, headless OvAbg females assume an egg pushing posture at every light stimulation (Fig. 2g and h), indicating that OvAbg brain projections do not contribute to this activation phenotype.

Next, we quantified egg expulsion during the activation protocol. We found that nearly half of the test flies expel an egg during the activation protocol, whereas control females do not expel eggs (Fig. 2i). Most of the eggs are expelled during the first stimulation period (Fig. 2j). In summary, activity of OvAbg consistently leads to an egg pushing posture, but does not always lead to egg expulsion.

Given that OvAbg neurons control a female specific behaviour, we asked whether these neurons are present in the male. Indeed, there are male OvAbg neurons, albeit fewer and with dimorphic projections (Supplementary Fig. 1l–n). Activation of OvAbg neurons in males always triggers two motor elements associated with copulation, abdomen curling and aedeagus extrusion, suggesting an analogous role of OvAbg neurons in male reproduction (Supplementary Fig. 1o and p).

To further investigate the role of OvAbg activity in egg-laying, we performed silencing experiments using the inwardly rectifier potassium channel Kir2.1^41^. For egg-laying experiments, females were paired with males on apple juice agar plates and monitored for two hours for copulation. The number of eggs laid by mated females over 24 h was nearly abolished by OvAbg silencing (Fig. 2k). Notably, we observed that 88.7% test and 75% control flies mated, showing that OvAbg neurons are not involved in female receptivity (Supplementary Fig. 1q). This result, together with the observation that flies survive and appear healthy with constitutive silencing of OvAbg neurons, indicates that they are specifically involved in egg-laying. To ascertain that egg-laying defect does not result from an impairment in egg production or ovulation, we dissected the reproductive system. We observed eggs jammed in the lateral oviducts in all test flies together with an excess of mature eggs in the ovaries (Fig. 2l and m), indicating that egg production and ovulation are not affected.

It is well established that octopamine has a key role in *Drosophila* egg-laying^28,42–45^, therefore we sought to explore the contribution of this modulator, as well as serotonin and dopamine, in the function of OvAbg neurons. Using an antibody to Tyrosine decarboxylase 2 (Tdc2), which is involved in the biosynthesis of octopamine, we did find an overlap with OvAbg neurons, albeit only in three neurons (n = 5) (Supplementary Fig. 2a–d). These octopaminergic OvAbg neurons represent a third of the dsx/Tdc2^+^ subset, previously shown to control egg-laying^42^. Unfortunately, we could not obtain genetic access to these neurons in order to investigate their role in egg-laying. We found no overlap of serotonin antibody with OvAbg neurons (Supplementary Fig. 2e and f). An intersectional approach between OvAbg and tyrosine hydroxilase (TH) expression revealed a small set of two to three dopaminergic neurons in the Abg (n = 4) (Supplementary Fig. 2h and i) and no expression in the brain (Supplementary Fig. 2g) or the reproductive system (n = 5) (Supplementary Fig. 2j). Optogenetic activation of OvAbg dopaminergic neurons did not elicit any egg-laying associated behaviour (data not shown). Constitutive silencing with Kir2.1 resulted in a small, but significant, decrease in the number of eggs laid (Supplementary Fig. 2k), which was not accompanied by an egg jamming phenotype (Supplementary Fig. 2l), suggesting a role of these neurons either in egg production or in ovulation. Previous studies indicated the involvement of dopamine in egg-laying exploration/site choice^46,47^. These results suggest that dopamine may also regulate egg-laying in a non-choice context.

In this series of experiments, we found a group of neurons of the egg-laying motor circuits which are directly involved in the execution of egg pushing and expulsion, and necessary for egg-laying. These Abg neurons provide a great entry point to address how different circuits execute egg deposition.

### Silencing OvAbg neurons disrupts all motor elements associated with egg-laying behaviour

**Figure 3:**
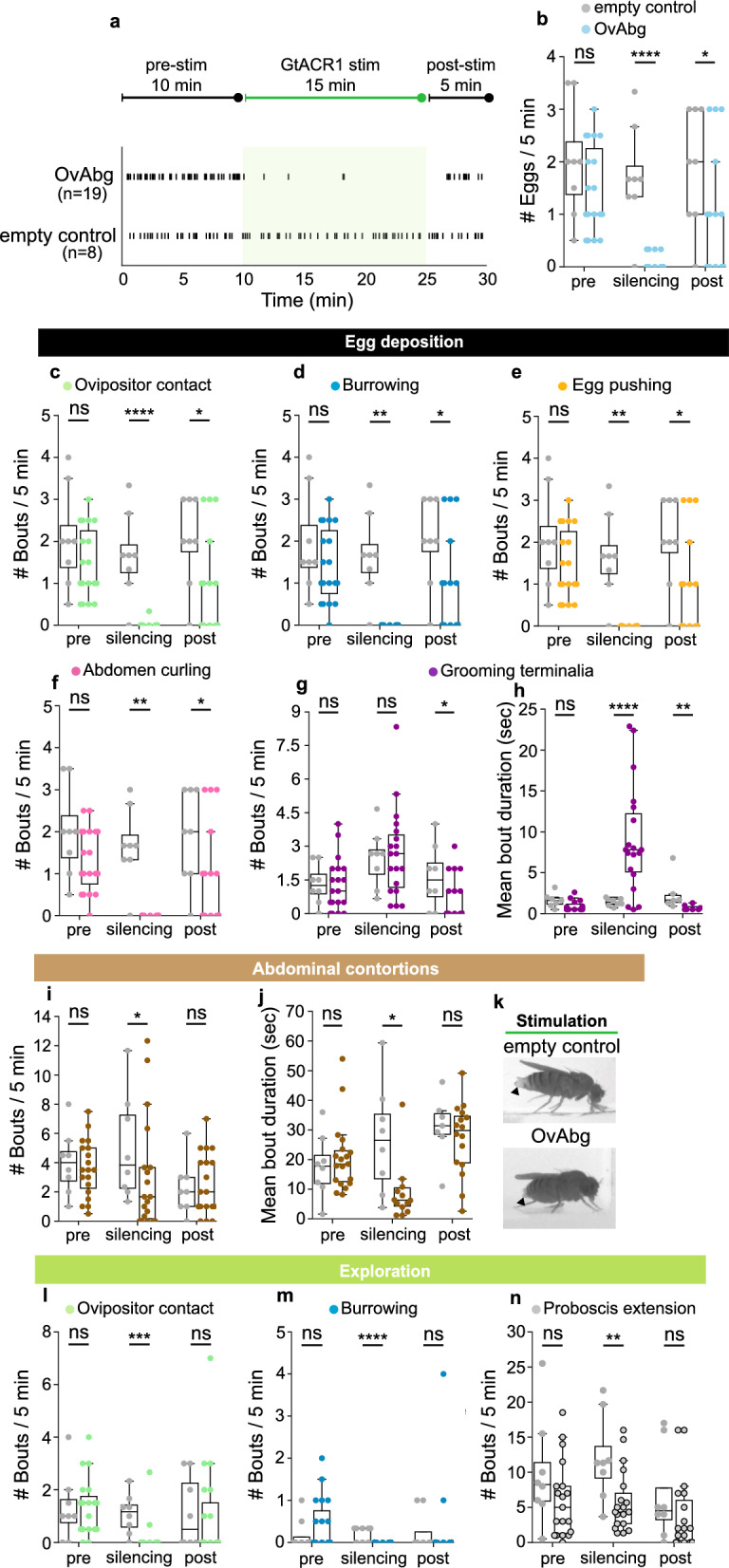
Silencing OvAbg neurons disrupts all motor elements associated with egglaying behaviour. (**a**) (top) GtACR1 silencing protocol scheme includes a pre-stimulation period (pre-stim; 10 min), a silencing period (stim; 15 min) followed by a post-stimulation period (post-stim; 5 min). (bottom) Raster plot shows the egg expulsion events for OvAbg silenced and control females throughout the 30 min of the experimental protocol. Green shaded area represents the stimulation period. The numbers in parentheses indicate the number of flies tested. (**b**) Number of eggs laid per fly during 5 min periods. (**c**–**g**) Egg deposition phase-associated motor elements and corresponding quantification of the number of behaviour bouts during 5 min periods. (**h**) Mean bout duration of grooming terminalia. (**i**) and (**j**) Number of abdominal contortions bouts during 5 min periods and the corresponding mean bout duration. (**k**) Frame snapshots of test (bottom) and control (top) flies displaying abdominal contortions during the silencing period. Besides displaying shorter bouts of abdominal contortions, silenced OvAbg flies also display less extended ovipositor extrusions during abdominal contortions in comparison with control flies that perform full ovipositor extrusions (arrowheads). (**l**–**n**) Exploration phase-associated motor elements and corresponding quantification of the number of behaviour bouts during 5 min periods. Box plots in (**b**–**j**) and (ln) indicate the median (middle line), 25th, 75th percentile (box) and 5th and 95th percentile (whiskers) as well as outliers (single points). n = 19 (OvAbg silenced) and 8 (control) flies in (**a**–**n**). Unpaired t-test and Mann–Whitney test applied in normally and non-normally distributed samples, respectively. ns p ≥ 0.05; *p < 0.05; **p < 0.01; ***p < 0.001; ****p < 0.0001 for comparisons against respective controls (gray datapoints) within each protocol phase.

We have shown that, upon activation of OvAbg neurons, a single egg deposition motor element—egg pushing—is induced and that OvAbg silenced females do not lay eggs. How do OvAbg silenced females behave? Do they perform all the behaviour elements, with the exception of egg pushing, or are other motor elements affected? To answer these questions, we used the anion channelrhodopsin, GtACR1^48^, for acute optogenetic silencing of OvAbg neurons. We analysed 15 min of light stimulation, as well as, 10 min pre- and 5 min post-stimulation (Fig. 3a). Acute silencing of OvAbg neurons severely affected the number of eggs laid during the stimulation, followed by a partial recovery in the post-stimulation period (Fig. 3a and b). Analysis of the behavioural elements showed that, with the exception of grooming, all egg deposition motor elements were abolished during stimulation and partially recovered post-stimulation (Fig. 3c–g). The expulsion of four eggs during the silencing period was executed without using most of the egg deposition motor programme (Supplementary Movie 4). The number of grooming bouts did not differ from control during silencing (Fig. 3g). However, the time the female spent grooming the terminalia was much larger in the test condition during silencing (Fig. 3h). Interestingly, both measures of grooming were reduced compared to control in the post-stimulation period, suggesting a rebound effect on circuits modulating grooming behaviour. The data, so far, showed a wide effect of silencing OvAbg neurons in all egg deposition elements.

We next analysed the other egg-laying phases. Abdominal contortions were reduced compared to control, both in number of bouts (Fig. 3i) and behaviour duration (Fig. 3j). Additionally, the intensity of the contortions and the extent of the ovipositor extrusion were reduced in test flies compared to controls, as exemplified in Fig. 3k. The behavioural elements of the exploration motor programme were likewise significantly reduced during silencing (Fig. 3l–n). In the case of proboscis extension, it should be noted that control flies increased proboscis extension bouts during the stimulation in relation to the pre- and post-stimulation periods (Fig. 3n, silencing) and, thus, the biological significance of the difference observed between control and manipulated flies during the stimulation period is not clear. Interestingly, both the abdominal contortions and exploration motor programmes fully recovered post-stimulation (Fig. 3i–n, post-stimulation), indicating that the regulation of these phases is simpler than that of the egg deposition motor programme, where the inhibitory effects of GtACR1 stimulation persisted (Fig. 3c–f, post-stimulation).

We have also tested the effect of silencing OvAbg neurons with Kir2.1 on the egg-laying motor programme, which we did not use as main result, because OvAbg Kir2.1 silenced females displayed a bloated abdomen (Supplementary Fig. 3a). However, we did confirm a reduction in the number of eggs laid (Supplementary Fig. 3b), which was accompanied by a reduction in all egg deposition motor elements (Supplementary Fig. 3c–g). Abdominal contortions and the exploration programmes showed little or no differences with the respective controls (Supplementary Fig. 3h–k), suggesting a possible compensation mechanism over long-term silencing.

Our findings show that acute silencing of OvAbg neurons affects all phases of egg-laying behaviour. We cannot rule out that some of the silencing effects we observed reflect an arrest on the egg-laying cycle (Fig. 1j) induced by the loss of egg pushing behaviour and inability to complete egg deposition, rather than a direct involvement of OvAbg neurons in all egg-laying phases.

### OvAbg/Gad1 neurons are involved in egg deposition

**Figure 4:**
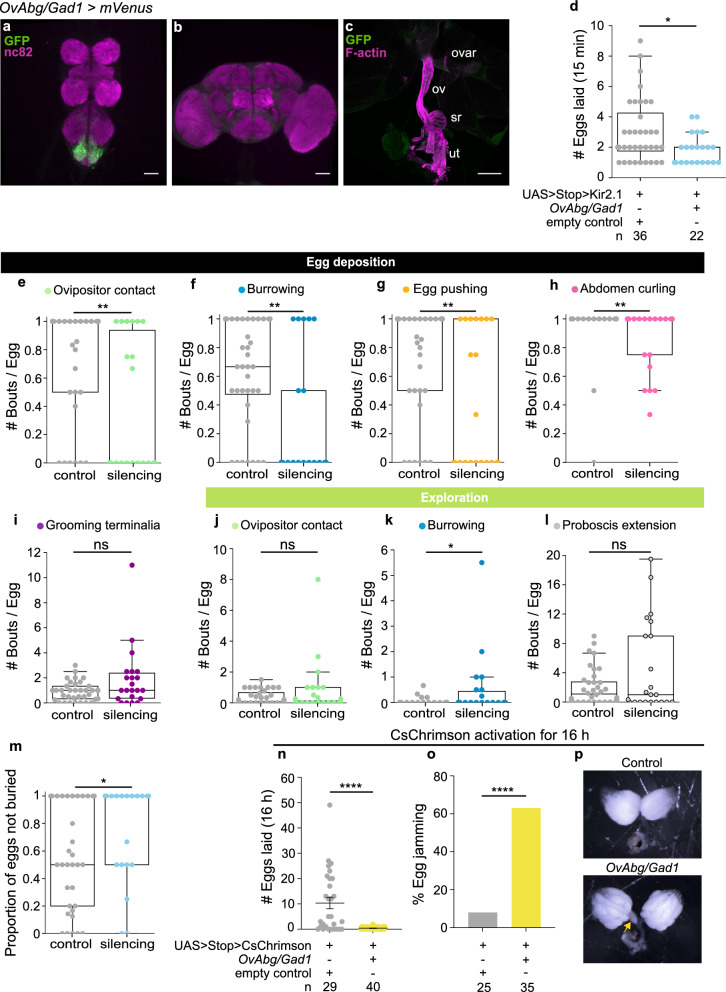
Activity of OvAbg/Gad1 neurons regulates egg-laying. (**a**–**c**) Confocal images of female VNC (**a**), brain (**b**) and reproductive system (**c**) of OvAbg/Gad1 neurons and corresponding innervations labelled with anti-GFP antibody (green) and nc82 for neuropil. Phalloidin, which binds F-actin, was used in (**c**) to visualize the muscle fibers. ovar: ovary; ov: oviducts; sr: seminal receptacle; ut: uterus. Anti-GFP antibody is targeting the fluorescent protein *Venus* from *OvAbg/Gad1-LexA* > *UAS* > *Stop* > *CsChrimson-mVenus* expressing flies. Scale bars, (**a**,**b**) 50 µm and (**c**) 200 µm. (**d**) Number of eggs laid by OvAbg/Gad1 silenced females during a 15 min period. (**e**–**i**) Egg deposition phase-associated motor elements and corresponding quantification of the number of behaviour bouts. (**j**–**l**) Exploration phase-associated motor elements and corresponding quantification of the number of behaviour bouts. In (**e**–**l**) the quantification of the number of behaviour bouts was normalized for the number of eggs laid per female and quantified for the entire video duration (15 min). m Proportion of eggs not buried per fly.Box plots in (**d**–**m**) indicate the median (middle line), 25th, 75th percentile (box) and 5th and 95th percentile (whiskers) as well as outliers (single points). n = 36 (control) and 22 (OvAbg/Gad1) females. n Number of eggs laid during the 16 h photoactivation with CsChrimson of OvAbg/Gad1 neurons. n = 29 (control) and 40 (OvAbg/Gad1) females. Bars indicate mean ± sem. (**o**) Percentage of activated OvAbg/Gad1 females with eggs jammed in the lateral oviducts. n = 25 (control) and 35 (OvAbg/Gad1) females. (**p**) (bottom) Image representing a reproductive system of an activated OvAbg/Gad1 female with one egg jammed (yellow arrow) in the lateral oviduct and a control (top) reproductive system with clear oviducts. Note that OvAbg/Gad1 activated females also have enlarged ovaries containing more mature oocytes (similar to OvAbg silencing egg jamming phenotype, see Fig. 2m). In (**d**–**n**) Unpaired t-test and Mann–Whitney test applied in normally and non-normally distributed samples, respectively. ns p ≥ 0.05; *p < 0.05; **p < 0.01; ****p < 0.0001 for comparisons against respective controls (grey datapoints). Fisher’s exact test, ****p < 0.0001 applied in (**o**).

To address how different neurons within the OvAbg population contribute to the execution of egg-laying, we used an intersectional approach where we genetically intersect OvAbg neurons with neurotransmitter driver lines^49^. The OvAbg/Gad1 neurons will be discussed here, while the intersections with *Cha* and *VGlut* will be discussed in the ensuing sections. OvAbg/Gad1 neurons (96 ± 10, n = 7) are mostly local interneurons with sparse and faint projections to other VNC ganglia (Fig. 4a). Co-staining with GABA antibody showed strong co-localization (75% overlap, Supplementary Fig. 4c and d). No projections of OvAbg/Gad1 neurons were observed in the brain (Fig. 4b) or the reproductive system (Fig. 4c).

We silenced OvAbg/Gad1 with Kir2.1, since a GtACR1 tool that allows this type of genetic intersection is not available. We video-recorded control and silenced flies for 15 min and annotated their behaviour. We found a decrease in the number of eggs laid during 15 min videos (Fig. 4d). The analysis of the egg-laying motor programme revealed a reduction in all egg deposition behavioural elements relative to the number of eggs laid, except for grooming terminalia (Fig. 4e–i, Supplementary Movie 5). Within the exploration programme, only burrowing was affected with a slight increase in the number of bouts (Fig. 4j–l). Abdominal contortions were not affected (Supplementary Fig. 4a).

Often egg-laying in *Drosophila* culminates in subterraneous egg expulsion. Since silencing OvAbg/Gad1 neurons reduces egg deposition behaviours, we investigated if silenced females had less eggs buried. Indeed, comparing the number of eggs not buried with the total number of eggs laid, we observed an increase in the number of eggs not buried during egg deposition (Fig. 4m), which is likely an outcome of the impairment in ovipositor contact and burrowing behaviours during this phase (Supplementary Movie 5). Interestingly, assessing the number of eggs laid by silenced OvAbg/Gad1 females with a different protocol, in which flies are younger and not egg-laying deprived, showed no egg laying reduction (Supplementary Fig. 4b, see “Methods”). It is not clear the exact cause of the differences in the phenotypes.

Next, we investigated whether activity of OvAbg/Gad1 drives egg-laying motor elements. Activating these neurons using the same stimulation protocol used to activate OvAbg neurons did not elicit any behaviour (data not shown). We postulate that, if OvAbg/Gad1 have a modulatory role, an effect on behaviour will only be visible with longer activations. To test this, we activated OvAbg/Gad1 neurons overnight (16 h activation). We observed that activation of OvAbg/Gad1 abolishes egg-laying (Fig. 4n). Dissection of the reproductive system at the end of the experiment revealed that the eggs are jammed at the lateral oviduct (Fig. 4o and p).

Our findings show that activity in OvAbg/Gad1 neurons supresses egg-laying and, thus, have the potential to gate egg-laying execution by other neurons in the OvAbg population. However, activity in OvAbg*/*Gad1 also seems to be required for a proper egg deposition bout. Given that OvAbg*/*Gad1 comprises approximately 96 neurons, it is possible that different neurons within this population mediate the contrasting silencing and activation phenotypes.

### OvAbg/Cha neurons are involved in egg pushing and expulsion

**Figure 5:**
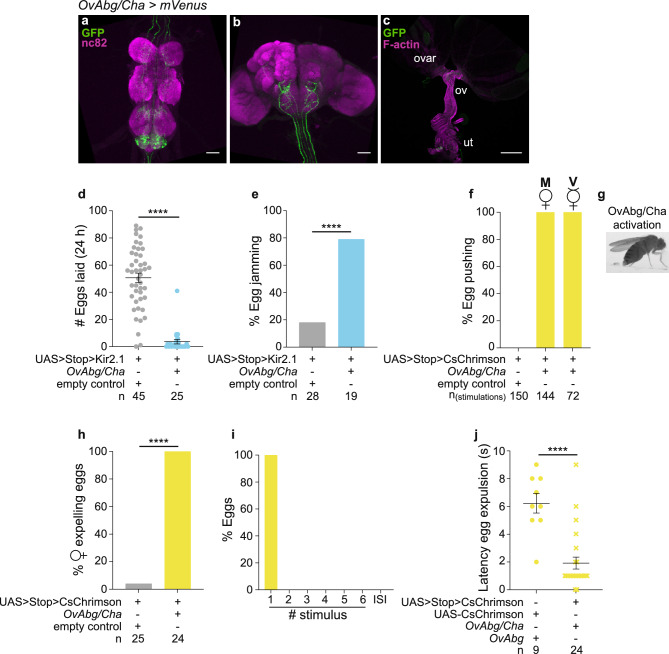
OvAbg/Cha neurons are involved in egg pushing and expulsion. (**a**–**c**) Confocal images of female VNC (**a**), brain (**b**) and reproductive system (**c**) showing OvAbg/Cha neurons and corresponding innervations stained with anti-GFP antibody (green) to reveal the membranes and nc82 for neuropil. Phalloidin, which binds F-actin, was used in (**c**) to visualize the muscle fibers. ovar: ovary; ov: oviduct; ut: uterus. Anti-GFP antibody is targeting the fluorescent protein *Venus* from *OvAbg*/*Cha-LexA* > *UAS* > *Stop* > *CsChrimsonmVenus* expressing flies. Scale bars, (**a**,**b**) 50 µm and (**c**) 200 µm. (**d**) Number of eggs laid in the 24 h after mating during inhibition of OvAbg/Cha neurons. n = 45 (control) and 25 (OvAbg/Cha) females. Bars indicate mean ± sem. Mann–Whitney test, ****p < 0.0001. (**e**) Percentage of females with eggs jammed in the lateral oviducts. n = 28 (control) and 19 (OvAbg/Cha) females. Fisher’s exact test, ****p < 0.0001. (**f**) Percentage of stimulation events in which OvAbg/Cha flies displayed an egg pushing-like posture. n = 150 (control), 144 (OvAbg/Cha mated females) and 72 (OvAbg/Cha virgin females) stimulations. (**g**) Frame snapshot of an activated OvAbg/Cha female displaying an egg pushing-like posture. (**h**) Percentage of activated OvAbg/Cha females expelling eggs. n = 24 (control) and 24 (OvAbg/Cha) females. Fisher’s exact test, ****p < 0.0001. (**i**) Percentage of eggs expelled by OvAbg/Cha females during stimulations and inter-stimulus intervals (ISI). n = 24 eggs. (**j**) Latency (seconds) to egg expulsion (period of time until the first egg expulsion after stimulus is ON) of OvAbg and OvAbg/Cha photoactivated females. n = 9 (OvAbg) and 24 (OvAbg/Cha) females. Bars indicate mean ± sem. Mann–Whitney test, ****p < 0.0001.

OvAbg/Cha neurons represent a large fraction of OvAbg neurons (Fig. 5a) that include projections to the brain (Fig. 5b), to the reproductive system (Fig. 5c) and to the abdominal wall (Supplementary Fig. 5a). Quantification of OvAbg/Cha neurons revealed that this intersection labels about the same number of neurons as the original line (OvAbg/Cha = 159 ± 12 (n = 6); OvAbg = 153). This is a surprising finding given that the OvAbg/Gad1 subset labels 96 neurons, raising the possibility of GABA co-expression among these OvAbg/Cha neurons. We did not see overlap between GABA staining and OvAbg/Cha labelling (Supplementary Fig. 5b and c), suggesting that this intersection labels non-OvAbg neurons, which may or may not contribute to the phenotypes described below.

Silencing OvAbg/Cha neurons with Kir2.1 led to a severe reduction in the number of eggs laid during 24 h (Fig. 5d) and a large fraction of these females displayed egg jamming in the lateral oviduct (Fig. 5e). These results showed a very similar phenotype to that observed when silencing all OvAbg neurons (Fig. 2k and l). The egg-laying behavioural elements were also affected in a similar fashion, with the exception of abdominal contortions and proboscis extension (OvAbg/Cha, Supplementary Fig. 5e–n and Supplementary Movie 6; OvAbg, Supplementary Fig. 3c–k).

Activation of OvAbg/Cha neurons, using the protocol shown in Fig. 2d, led to both virgin and mated females assuming an egg pushing posture each time the light is ON (Fig. 5f and g, Supplementary Movie 7), as observed when all OvAbg neurons are activated (Fig. 2d and e). No other egg-laying motor elements were triggered by OvAbg/Cha activation. Interestingly, quantification of females expelling eggs during the stimulation protocol revealed that all OvAbg/Cha females expelled one egg (Fig. 5h), in contrast to less than half of OvAbg females (Fig. 2i). Additionally, all eggs expelled during the stimulation protocol by OvAbg/Cha females were expelled during the first stimulus (Fig. 5i), whereas expulsion timing by OvAbg females during the stimulation protocol was variable, with females expelling eggs in the third and fifth stimulus, as well as in the interstimulus intervals (Fig. 2j). In summary, upon activation, if and when an egg is expelled, is variable for OvAbg, but not for OvAbg/Cha females.

We also quantified, within the 10 s stimulation bout, when the females expelled the egg. We found a striking difference between OvAbg and OvAbg/Cha females (Fig. 5j), with OvAbg females taking a longer time to expel the egg. The increased variability regarding when the egg is expelled during the stimulation protocol, and the increased latency to expel an egg upon light ON of the OvAbg females compared to the OvAbg/Cha females could result from: (1) differences in the levels of expression of UAS-CsChrimson used in the *OvAbg* line and UAS > stop > CsChrimson used in the *OvAbg/Cha* line; (2) contribution of non-OvAbg neurons labelled by the *OvAbg/Cha* line and/or (3) inhibition by the OvAbg/Gad1 neurons within the OvAbg population, meaning, activation of OvAbg neurons would encompass simultaneous activation of inhibitory OvAbg/Gad1 and OvAbg/Cha, which would result in conflicting information leading to variability and delay of the behavioural execution. The first hypothesis was discarded because quantification of fluorescence intensity between *OvAbg* and *OvAbg/Cha* lines showed no differences (Supplementary Fig. 5d). We favour the third hypothesis as the phenotype is consistent with the absence of GABAergic neurons in the *OvAbg/Cha* line, although we cannot rule out the second hypothesis.

Overall, the results indicate that OvAbg/Cha neurons are involved in the exploration and egg deposition phases and are sufficient for the execution of egg pushing leading to egg expulsion.

### OvAbg/VGlut neurons contribute to egg deposition initiation

**Figure 6:**
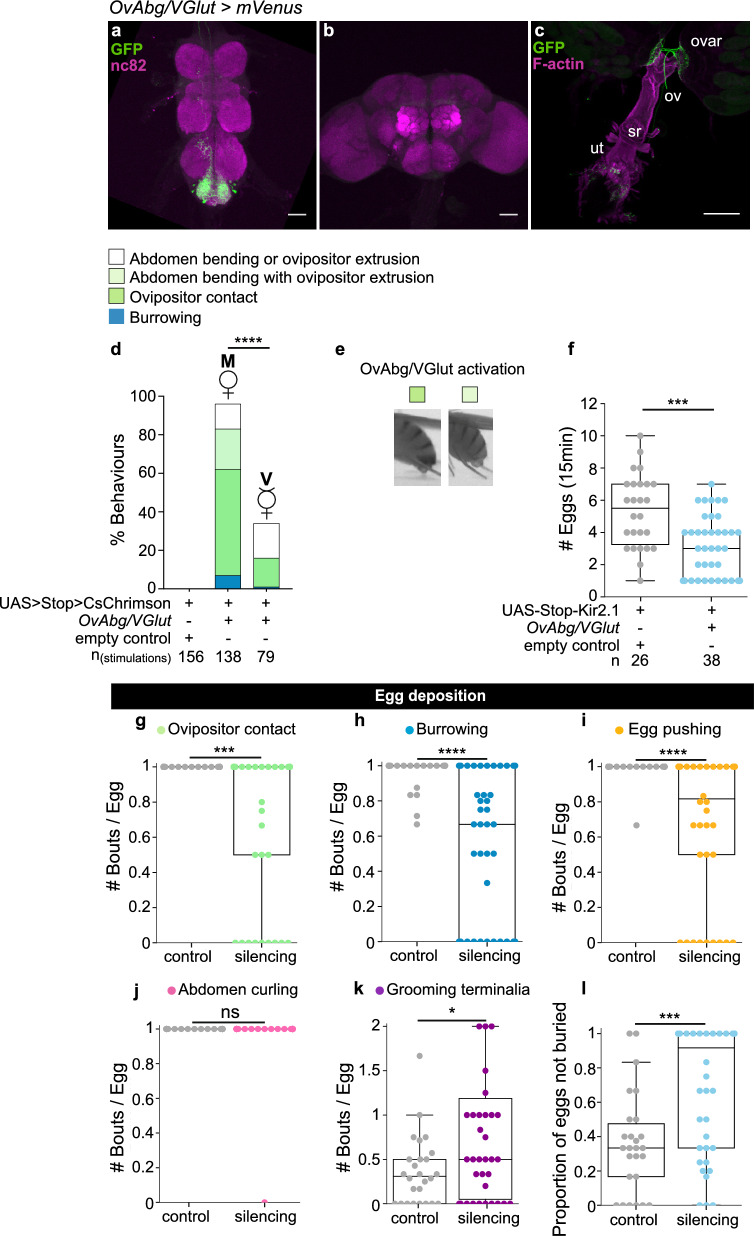
OvAbg/VGlut neurons contribute to egg deposition initiation. (**a**–**c**) Confocal images of female VNC (**a**), brain (**b**) and reproductive system (**c**) showing OvAbg/VGlutneurons and corresponding innervations stained with anti-GFP antibody (green) to reveal the anatomy and nc82 for neuropil. Phalloidin, which binds F-actin, was used in (**c**) to visualize the muscle fibres. ovar: ovary; ov: oviducts; sr: seminal receptacle; ut: uterus. AntiGFP antibody is targeting the fluorescent protein *Venus* from *OvAbg*/*VGlut-LexA* > *UAS* > *Stop* > *CsChrimson-mVenus* expressing flies. Scale bars, (**a**,**b**) 50 µm and (**c**) 200 µm. (**d**) Percentage of stimulation periods in which OvAbg/VGlut mated (M) and virgin (V) flies displayed ovipositor contact, burrowing, abdomen bending with ovipositor extrusion, and abdomen bending or ovipositor extrusion behaviours. n = 156 (control mated), 138 (OvAbg/VGlut mated) and 79 (OvAbg/VGlut virgin) stimulations. Fisher’s exact test, ****p < 0.0001. (**e**) Frame snapshots of an activated OvAbg/VGlut female displaying ovipositor contact (left) and abdomen bending with ovipositor extrusion (right) behaviours. Ovipositor contact behaviour implies contact between the ovipositor and the substrate, whereas this contact does not occur during abdomen bending with ovipositor extrusion (right). (**f**) Number of eggs laid over 15 min during inhibition of OvAbg/VGlut neurons. Mann–Whitney test, ***p < 0.001. (**g**–**k**) Egg deposition phase-associated motor elements and corresponding quantification of the number of behaviour bouts normalized for the number of eggs laid per female. (**l**) Proportion of eggs not buried per fly. n = 26 (control) and 38 (OvAbg/VGlut) flies in (**f**–**l**). Box plots in (**f**–**l**) indicate the median (middle line), 25th, 75th percentile (box) and 5th and 95th percentile (whiskers) as well as outliers (single points). Unpaired t-test and Mann–Whitney test applied in normally and non-normally distributed samples, respectively. ns p ≥ 0.05; *p < 0.05; ***p < 0.001; ****p < 0.0001 for comparisons against respective controls (gray datapoints).

We found that OvAbg/VGlut neurons are the smallest functional group composed by 14 neurons (± 4, n = 4). We observed projections in the VNC, some unilateral and some bilateral, and occasionally we observed projections in the brain (Fig. 6a and b). This group of neurons also projects to the lateral oviducts, distal uterus (Fig. 6c) and to the abdominal wall (Supplementary Fig. 6a).

Optogenetic activation of OvAbg/VGlut neurons in mated females, using the protocol depicted in Fig. 2d, triggered ovipositor contact-related behaviours (Supplementary Movie 8). We found that 55% of the stimulations led to ovipositor contact behaviour (Fig. 6d and e, bright green box), while abdomen bending with ovipositor extrusion not touching the substrate (Fig. 6e, pale green box) was identified in 21% of the stimulation periods (Fig. 6d). Additionally, in a small fraction of the stimulations (7%) flies displayed burrowing behaviour accompanying ovipositor contacts (Fig. 6d). Finally, we observed in 14% of stimulations, flies either extruded the ovipositor with straight abdomen or bent the abdomen without extruding the ovipositor. Control flies did not display any of these behaviours during light ON (Fig. 6d).

To test possible effects of egg accumulation due to the egg-laying deprivation protocol, we repeated the activation experiment with deprived and non-deprived females. We observed similar activation phenotypes between the two conditions (Supplementary Fig. 6b). Interestingly, we found that virgin and mated OvAbg/VGlut females display different behavioural phenotypes upon activation. In virgins, 66% of the stimulations do not evoke any behaviour (Fig. 6d). Ovipositor extrusion or abdomen bending were observed in 18% of the stimulations. Ovipositor contact was identified in only 15% of the stimulations and burrowing behaviour was residual (1% stimulations) (Fig. 6d). This result suggests that the behavioural output of OvAbg/VGlut neurons is modulated by the mating status.

In the first section of this study, we characterised ovipositor contact and burrowing as motor elements displayed by mated females in the exploration and egg deposition phases. The phenotype of OvAbg/VGlut activation provides evidence for a role of OvAbg*/*VGlut in controlling these behaviours, which could be specific to one of those phases, or be implicated in both. To test this, we investigated in detail the egg-laying behaviour in OvAbg/VGlut females silenced with Kir2.1. Silencing OvAbg/VGlut neurons reduced, but did not abolish, the number of eggs laid (Fig. 6f), suggesting that this neuronal subset is not necessary for egg-laying, in contrast to OvAbg/Cha group. Analysis of the behavioural elements showed that OvAbg/VGlut silencing affects egg deposition behaviours up to egg expulsion: ovipositor contact, burrowing and egg pushing (Fig. 6g,h,i). Silenced OvAbg/VGlut females displayed fewer bouts of each behaviour per egg, although we still observed manipulated flies that performed these behaviours at levels comparable to control. This reduction in the expression of motor elements that precede egg expulsion results in abnormal initiation of the egg deposition bout (Supplementary Movie 9). The post-egg expulsion behaviours—abdomen curling and grooming—were not reduced (Fig. 6j and k). In fact, grooming was slightly increased which may be associated with the deficient egg deposition (Fig. 6k). Indeed, the increased grooming in test females is specifically associated with the egg deposition events, rather than a generalised increase in grooming terminalia throughout the video (Supplementary Fig. 6c and d). The abdominal contortions phase was not affected (Supplementary Fig. 6e).

Glutamate has been shown to be involved in oviduct contractions^29^, which presumably occur during the contortions phase. Our results indicate that there is a distinct group of glutamatergic neurons, not labelled by OvAbg, involved in oviduct contractions. The exploration phase, which shares two motor elements with the egg deposition phase (ovipositor contact and burrowing) was not affected (Supplementary Fig. 6f–h). We also investigated if silenced females had less eggs buried. Indeed, we observed that OvAbg/VGlut silenced females had less eggs buried in the agarose substrate (Fig. 6l) in comparison to control flies.

In summary, the activation and silencing results strongly suggest that OvAbg/VGlut neurons are involved in the initiation of the egg deposition motor sequence. Although OvAbg/VGlut neurons are not necessary for egg-laying, egg-laying is less efficient when these neurons are due to impaired egg deposition initiation. Furthermore, similar to the manipulation of OvAbg/Gad1 neurons, OvAbg/VGlut neurons play a role in progeny survival as their silencing leads to fewer eggs buried and, therefore, increased exposure to predation and adverse climate conditions.

### oviDNs neurons functionally connect to OvAbg neurons

Next, we addressed the connectivity of OvAbg neurons with central neurons using GFP reconstitution across synaptic partners (GRASP), which reveals membrane contact between two sets of neurons^50,51^. We sought to identify connectivity with the egg-laying command neurons (oviDNs), recently described to elicit the egg deposition behavioural sequence, and arborizing in the abdominal ganglion^52,53^ (for anatomy, see Supplementary Fig. 7a and b).

**Figure 7:**
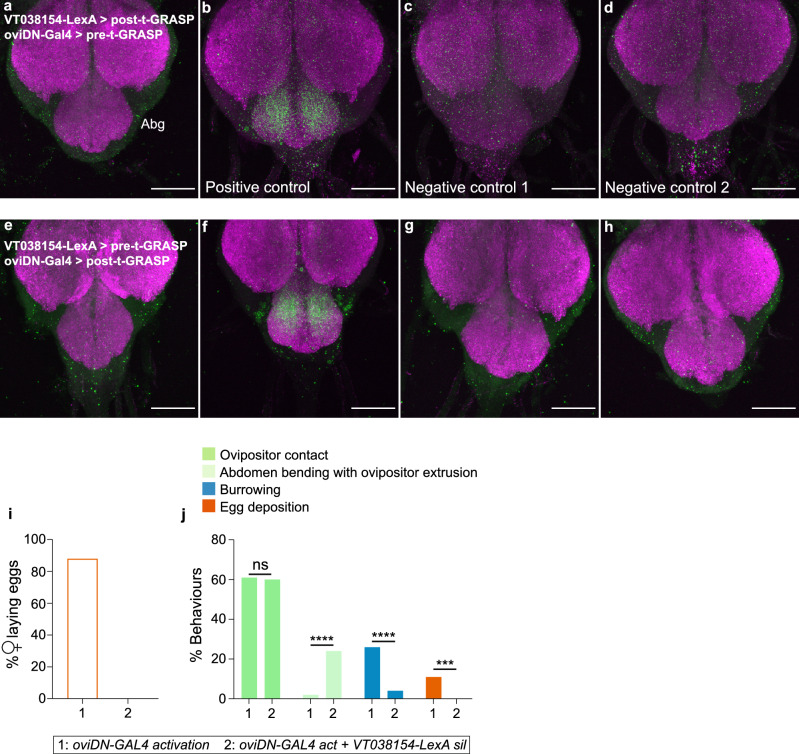
OvAbg neurons connect to oviDNs neurons. a-h Confocal images of the abdominal ganglion (Abg) showing t-GRASP between VT038154-LexA and oviDN-Gal4, (**a**–**d**) VT038154-LexA expressing LexAop-post-t-GRASP and oviDN-Gal4 driving expression of UAS-pre-t-GRASP. Positive control: VT038154-LexA expressing LexAop-post-t-GRASP and nSyb-Gal4 driving expression of UAS-pre-t-GRASP. Negative control 1: VT038154-LexA expressing Lexaop-post-t-GRASP and UAS-pre-t-GRASP. Negative control 2: oviDN-GAL4 expressing Lexaop-post-t-GRASP and UAS-pre-t-GRASP. (**e**–**h**) VT038154-LexA expressing LexAop-pre-t-GRASP and oviDN-Gal4 driving expression of UAS-post-t-GRASP. Positive control: VT038154-LexA expressing LexAop-pre-t-GRASP and nSyb-Gal4 driving expression of UAS-post-t-GRASP. Negative control 1: VT038154-LexA expressing Lexaop-pre-t-GRASP and UAS-post-t-GRASP. Negative control 2: oviDN-GAL4 expressing Lexaop-pre-t-GRASP and UAS-post-t-GRASP. Scale bars, 50 µm. (**i**) Percentage of females laying eggs when oviDNs neurons are activated by CsChrimson in combination with VT038154-LexA silencing neurons with Kir.2.1 (treatment 2). The control is oviDNs activation only (treatment 1). sil: silencing; act: activation. n = 17 (control) and 17 (test) females. (**j**) Percentage of stimulation periods in which control (treatment 1) and ‘oviDN-GAL4 act + VT038154-LexA sil’ (treatment 2) flies displayed ovipositor contact (bright green), abdomen bending with ovipositor extrusion (pale green), burrowing (blue) and egg deposition (red) behaviours. Note that in this analysis ‘egg deposition’ represents the egg deposition behavioural sequence leading to egg expulsion, which includes ovipositor contact, burrowing and egg pushing behaviours. n = 89 (control) and 91 (test) stimulations. Fisher’s exact test, ns p ≥ 0.05; ***p < 0.001; ****p < 0.0001 for comparisons against treatment 1.

To test GRASP with oviDNs, we generated a LexA version of one of the split partners (*VT038154-LexA*) that make up the *OvAbg* line, allowing independent expression from oviDNs. This line has a similar expression pattern in the Abg to the *OvAbg* line, albeit with additional expression in other regions of the nervous system (Supplementary Fig. 7c–e). Verification of the activation phenotype using the same protocol used for OvAbg (Fig. 2d), revealed a less striking phenotype, where females bend the abdomen and extrude the ovipositor without contacting the substrate or expelling eggs (Supplementary Fig. 7f,g). To our surprise GRASP revealed no connections between VT038154-LexA and oviDN populations, while connections were clearly visible when using a pan-neuronal positive control (Fig. 7a–h). The results indicate that the abdominal ganglion has multiple layers of connectivity even though it is a small region.

To test the role that VT038154-LexA neurons play upon oviDNs command, we activated oviDNs using CsChrimson while silencing VT038154-LexA using Kir2.1. First, we confirmed that activation of oviDNs elicits all motor elements leading to an egg expulsion in most flies (Fig. 7i, treatment 1). Since egg expulsion occurs only once during the stimulation protocol, the egg deposition behavioural sequence was observed in 11% of the stimulations (Fig. 7j, red bar, treatment 1). In the stimuli without egg expulsion, oviDNs activation led to ovipositor contact or burrowing (which includes ovipositor contact) (Fig. 7j, bright green and blue bars, respectively, treatment 1). oviDNs activation in parallel with VT038154-LexA silencing revealed a selective interaction between these groups. We observed abrogated egg deposition (Fig. 7i, treatment 2; Fig. 7j, red bar, treatment 2). Curiously, burrowing behaviour was reduced (Fig. 7j, blue bar, treatment 2), while ovipositor contact was not affected (Fig. 7j, bright green bar, treatment 2). This result indicates that these two very similar behaviours require different neurons for their execution. We also observed abdomen bending with ovipositor extrusion (not touching the substrate), which was not observed in oviDNs activation. Given that the proportion of this acquired behaviour is similar to the loss of burrowing, it is tempting to speculate that abdomen bending with ovipositor extrusion represents a failed burrowing execution (compare blue and pale green bars, Fig. 7j).

In summary, our findings show that the execution of the egg-laying command by oviDNs relies on activity of OvAbg neurons though they are not synaptic partners.

## Discussion

Egg-laying is an excellent model for exploring the architecture of lower motor control as it is a complex behaviour constituted by different phases, each with a specific behavioural repertoire. In our observations, complementary to Yang et al*.*^2^, egg-laying of wild type flies is structured in three phases—egg deposition, abdominal contortions and exploration. We showed that the egg deposition phase is always followed by abdominal contortions, which may be followed by a new egg deposition event or by an exploration period. In this work, the exploration phase is defined by the ovipositor contact with the substrate, burrowing and the proboscis extension. In our assays, we see that the exploration phase is not obligatory. Two factors may explain this observation: (1) there may be exploration that does not include the motor elements we considered and rather flies use the mechanosensory and chemosensory information of the legs; (2) we use very small arenas with a restricted space for exploration and without complex sensory cues. This feature may allow a quick spatial and sensory recognition of the environment making exploration less frequent. Future work on the features of exploration and site selection should use more complex arenas and environments, and consider kinematic analysis. This detailed description of the behaviour provided a framework to interrogate the underlying neuronal substrates.

We identified three populations of OvAbg neurons in the abdominal ganglion with diverse contributions to the egg deposition phase. All three populations innervate similar areas in the abdominal ganglion, allowing potential connections between the three populations as part of an egg deposition circuit. The genetic make-up of these populations would predict a correspondence between the driver line used and the neurotransmitter expression. Unfortunately, we could not always control for the specificity of neurotransmitter expression, as Cha and VGlut antibodies label the cell bodies poorly and it is difficult to attribute colocalization to neuronal processes especially when more than a pair of neurons is labelled. OvAbg/Cha neurons are required for exploration and egg deposition phases, but their activation leads specifically to egg pushing followed by egg expulsion. OvAbg/VGlut neurons are involved in the initiation of egg deposition. Interestingly, the initial motor elements of egg deposition that are shared with exploration—ovipositor contact and burrowing—were not affected in the exploration phase, suggesting different control mechanisms for the same motor elements during different phases. Our findings suggest that OvAbg/VGlut and OvAbg/Cha populations collaborate in the execution of egg deposition. OvAbg*/*Gad1 neurons also participate in egg deposition as shown by the silencing experiments. On the other hand, our data shows that prolonged activation of this subset supresses egg-laying, indicating that activation pattern and strength are important for OvAbg*/*Gad1 function. Additionally, unlike the other two subsets, activation of OvAbg*/*Gad1 neurons does not elicit a postural change, which is consistent with a regulatory role, rather than executive, on egg-laying behaviour. We speculate that OvAbg*/*Gad1 neurons provide local suppression of egg-laying when negative egg-laying cues arise.

The abdominal ganglion egg deposition circuit is poised to receive commands from the brain. We showed that the execution of an egg-laying command by oviDNs relies on OvAbg neurons, though they are not synaptic partners. This observation is in line with the finding in the larval connectome that descending neurons target a small fraction of premotor circuit interneurons in the nerve cord^54^.

Egg-laying is essentially performed by mated females though virgin females may deposit unfertilized eggs residually. Activation of OvAbg*/*VGlut neurons leads to fewer events of egg deposition initiation in virgin females when compared to mated females, thus suggesting that mating status modulation of egg-laying is occurring locally at the abdominal ganglion in addition to the modulation in the brain^52,55,56^. Local modulation may result from direct octopaminergic modulation^42^ or in downstream targets.

Our findings provide a detailed description of egg-laying. We described the different motor elements, their participation in different egg-laying phases and how flies transition from one phase to the next. This description facilitates the goal of linking a complex behaviour—egg-laying—with its neuronal underpinnings. Using our framework, we present insights into the logic of egg deposition circuits. This work serves as a stepping stone to dissect ascending and descending communication with the brain and reproductive tract, to extend neuronal dissection and connectivity of egg-laying populations, and to address mechanisms of local mating status modulation of egg-laying.

## Methods

### Fly stocks and husbandry

See Supplementary Tables 1 and 2 for genotypes of *Drosophila* used in this study. Fruit flies *D. melanogaster* were raised in standard cornmeal-agar medium, using Vienna food recipe (In 1 L of water: 80 g molasses-barley malt, 22 g beet syrup, 80 g corn flour, 18 g granulated yeast, 10 g soy flour, 8 g agar–agar, 8 mL propionic acid,12 mL 15% nipagin, 35 mL Bavistin), at 25 °C and 70% relative humidity in a 12 h dark:12 h light cycle. Detailed information on fly housing and age for each experiment are indicated in the relevant section.

### Construction of transgenic lines

The fragment VT038154 was amplified from the CH321-73E23 BAC clone using the following primers: left primer-GCAGCTAACCTTCCACTCGGCAATC; right primer-ATGAAGGCCAGCCAGCGGATATTG and cloned into pCR™8/GW/TOPO™ to create the entry clone. VT038154-Entry Clone was then recombined into pBPnlsLexA_p65Uw to create the VT038154-LexA expression vector by LR Gateway recombination. The Plasmid was injected into y1w 67c23; P{CaryP}attP40 flies^57^ by adapting a protocol from Kiehart et al.^58^.

### Immunohistochemistry

Adult brains, VNCs, reproductive systems and abdomen cuticles were dissected in cold phosphate-buffered saline (PBS) and immediately transferred to cold PFA 4% in PBL (PBS with 0.12 M Lysine) and fixed for 30 min at RT, washed three times for 5 min in PBT (PBS with 0.5% Triton X-100) and blocked for 30 min at RT in 10% normal goat serum in PBT (Sigma, cat# G9023). Samples were incubated with the primary antibodies in blocking solution, for 72 h at 4 °C. The following primary antibodies were used: rabbit anti-GFP 1:1000 (Molecular Probes, cat#A11122), chicken anti-GFP 1:1000 (abcam, ab13970), rabbit anti-GFP for GRASP 1:100 (Anti-GFP Tag Abfinity, ThermoFisher, G10362), rabbit anti-Tdc2 1:200 (abcam, ab128225), rabbit anti-5HT 1:1000 (Sigma, cat# S5545), rabbit anti-GABA 1:100 (Sigma, cat# A2052), mouse anti-nc82 1:10 (Developmental Studies Hybridoma Bank, cat# AB_2314866), rabbit anti-DsRed 1:1000 (Takara, cat# 632496). Samples were washed three times for 5 min in PBT and incubated in Alexa Fluor 488 or 594 secondary antibodies 1:500 (Invitrogen) for 72 h at 4 °C. To counterstain the female reproductive system, Alexa 594-conjugated phalloidin (Molecular Probes, cat# A12381) was used. Samples were washed three times for 5 min in PBT and mounted in VectaShield medium (Vector Laboratories, cat#H-1000). Images were acquired on a Zeiss LSM 980 inverted laser scanning confocal microscope using a 25X immersion objective (Zeiss) for the brains/VNCs and a 10× objective (Zeiss) for the reproductive systems and abdomen cuticles. After acquisition, colour levels were adjusted using Fiji^59^ for optimal display.

### Preparation of flies to be assayed

Low fly density crosses (10–15 virgin females × 5 males per bottle) were used in all experiments for rearing flies with the appropriate genotype. In order to maximize the occurrence of egg deposition events during behaviour experiments, we followed the egg-laying deprivation protocol described by Yang et al.^60^ in all experiments, except in the 24 h egg-laying assays. Briefly, groups of 5–7 virgin females per vial of the appropriate genotypes and 2–3 Canton S males (for mating) were collected into normal food vials with the exception of optogenetic experiments in which flies were housed in normal food containing all-trans-Retinal (Sigma, R2500) (all-trans-Retinal concentrations used: 0.2 mM for CsChrimson activation and 0.4 mM for GtACR1 silencing). In contrast with the original protocol^60^, yeast paste was not added to the food. Flies were left in the vials for 4 to 7 days at 25 °C and 70% relative humidity. Behavioural assays were performed within that 4–7 days’ time window. In the ‘not egg-laying deprived’ condition in Supplementary Fig. 6b, females were handled following the protocol mentioned above, except that they were tested 2 days after mating.

### Behavioural assays

#### 24 h egg-laying assay

Single virgin females were gently aspirated and transferred to 35 mm Petri dishes of 10 mm of height (Thermo Fisher Scientific) coated with apple agar (750 mL water, 250 mL apple juice, 19,5 g agar, 20 g sugar, 10 mL 10% nipagin) and incubated with a naive Canton S male for 2 h under constant observation to check for mating occurrence. Plates where mating did not happen were discarded. Flies aged 4–7 days were kept in the plate for 24 h before eggs were counted. After egg counting, the female´s reproductive system was dissected to measure egg jamming.

#### Egg-laying arena and substrate

Custom made small rectangular-shaped arenas with 2 chambers were designed to allow recording of 2 flies simultaneously. Each chamber measures 1.8 (H) × 1.2 (L) × 0.3 (D) cm. During behavioural assays, the chambers were partially filled with the egg-laying substrate, which in this study was always 1% agarose (SeaKam^®^ LE Agarose, cat# 50004) diluted in distilled water (Milli-Q^®^ Water Purification Systems Merk). Flies had a free walking space of 1.5 × 0.7 × 0.3 cm in the chamber for egg-laying. The base and the lid of the arena were made of white opaque and transparent acrylic, respectively.

#### Detailed behaviour

To analyse the motor elements associated with egg-laying behaviour, females were collected soon after eclosion and housed in groups following the egg-laying deprivation protocol described above. Aged 5–8 days females were tested. Flies were gently aspirated into the egg-laying arena and behaviour was recorded at 20 frames per second during 45 min for Canton S (Fig. 1) and during 15 min for OvAbg (Fig. 3 and Supplementary Fig. 3) and OvAbg sub-groups silencing experiments (Figs. 4, 6 and Supplementary Fig. 5). The same fly handling procedure was performed for optogenetic experiments, in which egg-laying motor elements were analysed (for more detailed information, see the “Optogenetics” section).

#### Optogenetics

For all experiments using CsChrimson, except in the 16 h egg-laying assay (Fig. 4n–p), the stimulation protocol included a 1 min baseline period followed by 6 repetitions of 10 s red-light stimuli with a power of 4.40 mW/cm^2^ and 20 s interval between stimuli. In the OvAbg headless females’ photoactivation (Fig. 2g), the head was gently cut using dissection forceps (Dumont #55 Forceps, 11295-51) under CO_2_ anaesthesia. Flies were transferred to the egg-laying arena and allowed to recover from this procedure for 5–10 min before photoactivation. In the 16 h photoactivation egg-laying assay (Fig. 4n–p), mated females were transferred to the apple agar plates (described in the “24 h egg-laying assay” section). The stimulation protocol included constant red-light with a power ranging 4.19–4.85 mW/cm^2^ during 16 h. At the end of this period, the eggs were counted and the reproductive system was dissected to measure egg jamming. For the silencing experiment using GtACR1 (Fig. 3), the stimulation protocol included a pre-stimulation period that lasted for 10 min, followed by constant green-light stimulation with a power of 5–6.23 mW/cm^2^ during 15 min and a post-stimulation period of 5 min. Videos were recorded at 20 frames per second, except in the *OvAbg* line activation experiments (Fig. 2e–j), in which we used 15 frames per second.

#### Image capture

Flies were filmed using a camera mounted above the arena (Teledyne Flir Flea3 FL3-U3-13S2M equipped with a 16 mm fixed focal length lens (Edmund Optics)) and with a resolution of 1328 × 1048 pixels. For illumination, an infrared light using a 940 nm LED strip (SOLAROX) and a Hoya 49 nm R72 infrared filter (to reduce interference of visible light) were used. An electronic HARP LED array interface v1.3 and a HARP LED array v2.0 developed by the Champalimaud Foundation Hardware Platform were used to evoke a high-powered 610 nm (CsChrimson activation) and 527 nm (GtACR1 silencing) light. Bonsai software^61^ was used to trigger the optogenetics stimuli and to acquire the videos as avi files.

### Quantification and statistical analysis

#### Quantification of mVenus fluorescence intensity

Ventral nerve cords of 3–6 days old females were dissected in cold PBS and fixed for 30 min in 4% PFA. Images of the abdominal ganglia were acquired all on the same day, with the same setting in a Zeiss LSM 980 inverted laser scanning confocal microscope. The mean gray value for 10 cell bodies per abdominal ganglion was obtained, blind to the genotype, using Fiji^59^ (Supplementary Fig. 5d).

#### Data processing

After movies were acquired, the in-house developed software Python VideoAnnotator was used to manually annotate the time and duration of all egg-laying motor elements under analysis. Annotations were done for the total duration of the video in all experiments.

#### Quantification of behaviours

Data and statistical analysis were performed using custom Python scripts in Figs. 1b–j, 3, 4d–m and 6f–l; Supplementary Figs. 3b–k, 4a, 5e–n, 6c–h. All the other analysis were performed using Prism9 (GraphPad Software, La Jolla, CA).

The inter-egg expulsion intervals (Fig. 1d) were calculated as

$$\begin{aligned} {\text{Inter-}}{\text{egg expulsion intervals }} &= {\text{ first frame of egg expulsion bout }} \\ & \quad - {\text{ last frame of previous egg expulsion bout }}\left( {\text{per fly}} \right) \end{aligned}$$

The number of eggs per 5 min (Fig. 3b) was calculated as

$$\# {\text{ Eggs/5 min }} = \, \left( {{\text{sum }}\# {\text{ egg expulsions}}} \right){\text{/5 minutes }}\left( {\text{per fly}} \right)$$

The number of behaviour bouts per 5 min (Fig. 3c–g,i,l–n) was calculated as

$$\# {\text{ Bouts/5 min }} = \, \left( {{\text{sum }}\# {\text{ behaviour frames}}} \right){\text{/5 minutes }}\left( {\text{per fly}} \right)$$

The number of behaviour bouts per egg (Figs. 4e–l, 6g–k, Supplementary Figs. 4a and 6e–h) was calculated as

$$\# {\text{ Bouts/Egg }} = \, \left( {{\text{sum }}\# {\text{ behaviour frames}}} \right){\text{/total }}\# {\text{ egg expulsions }}\left( {\text{per fly}} \right)$$

The mean bout duration (Fig. 3h and j) was calculated as

$${\text{mean bout duration }} = \, \# {\text{ behaviour frame duration }}\left( {\text{s}} \right){\text{/total }}\# {\text{ behaviour bouts }}\left( {\text{per fly}} \right)$$

where the behaviour frame duration is given by the last frame subtracted to the first frame of each behaviour bout converted to seconds.

The proportion of eggs not buried (Figs. 4m and 6l) was calculated as

$${\text{Proportion of eggs not buried }} = \, \left( {{\text{sum }}\# {\text{ eggs not buried}}} \right){\text{/total }}\# {\text{ eggs }}\left( {\text{per fly}} \right)$$

Egg expulsion bouts in which it was not possible to determine whether the egg was buried or not were excluded from this analysis.

The percentage of behaviour displayed by flies during photoactivation (Figs. 2e–g, 5f, 6d, 7j, Supplementary Figs. 6b and 7f) was calculated as

$$\% {\text{ Behaviour }} = \, \left( {{\text{sum }}\# {\text{ stimuli with behaviour}}} \right){\text{/total }}\# {\text{ stimuli}}$$

The percentage of females laying eggs (Figs. 2i and 5h, 7i) was calculated as

$$\% {\text{ Females laying eggs }} = \, \left( {{\text{sum }}\# {\text{ females that laid eggs}}} \right){\text{/total }}\# {\text{ of females}}$$

The percentage of eggs laid during the photoactivation protocol (Figs. 2j and 5i) was calculated as

$$\% {\text{ Eggs }} = \, \left( {{\text{sum }}\# {\text{ eggs laid on each stimulus or ISI}}} \right){\text{/total }}\# {\text{ eggs}}$$

The latency for egg expulsion (Fig. 5j) was calculated as

$$\begin{aligned} {\text{Latency egg expulsion }} & = {\text{ first frame corresponding to egg expulsion event }} \hfill \\ & \quad -{\text{ first frame corresponding to the stimulus initiation }}\left( {\text{per fly}} \right) \hfill \\ \end{aligned}$$

The product was converted to seconds.

The percentage of eggs jammed (Figs. 2l, 4o and 5e) was calculated as

$$\% {\text{ Eggs jammed }} = \, \left( {{\text{sum }}\# {\text{ reproductive systems with eggs in the oviducts}}} \right){\text{/total }}\# {\text{ reproductive systems}}$$

To investigate the egg-laying phases transition probability (Fig. 1j), we first choose an egg-laying motor element to represent each phase: egg expulsion represented the egg deposition phase, abdominal contortions represented the abdominal contortions phase and ovipositor contacts that do not progress to egg expulsion represented the exploration phase. Proboscis extension and burrowing behaviours were not considered for the exploration phase transition analysis because: (1) proboscis extension displays a continuous occurrence during egg-laying behaviour (see Fig. 1i); (2) proboscis extension behaviour is not exclusive to egg-laying, being also displayed in other behavioural contexts (ex: feeding); (3) burrowing behaviour is displayed along with ovipositor contact but with lower frequency (see Fig. 1i). We calculated for the total video duration (45 min), the likelihood of all transitions between phases by using a first order Markov Chain analysis^62^. Phases transitions were identified as changes in the selected behavioural patterns for each phase.

To calculate the probability of the egg-laying behaviours around egg expulsion (Fig. 1f–i and Supplementary Fig. 6c and d) we aligned all the egg expulsion events of all flies and we counted how many behaviours were occurring in each of the 1200 frames preceding and following the end of egg expulsion. We then normalized the counts over the total number of egg expulsions. To represent the egg expulsion event, the last frame for each event was selected. We excluded from the analysis egg expulsion events that are less than 1200 frames from the start or end of the video.

#### Statistical analysis

Boxplot in Fig. 1c indicates the median flanked by the 25th and 75th percentiles (box) and whiskers showing the maximum and minimum values. In all the other boxplots, the whiskers represent the 5th and 95th percentiles. Error bars, when shown, are mean ± standard error of the mean (sem). Prior to statistical testing, normality across all individual experiments was verified using the Shapiro's Test, D'Agostino's Test, Anderson–Darling test and Kolmogorov–Smirnov in Prism9 analysis. Only Shapiro´s and D’Agostino’s tests were used in Python analysis. To assess the data variance homogeneity, Levene’s and Bartlett’s tests were used for non-normally and normally distributed data, respectively. In all experiments, we performed pairwise comparisons. Mann–Whitney test was used in non-normally distributed data. t-test was used in normally distributed data and the t-test with Welch´s correction was applied when data had unequal variances. Fisher´s exact test was used in contingency tables. The p-value is provided in comparison with the control and indicated as * for p < 0.05, ** for p < 0.01, *** for p < 0.001, **** for p < 0.0001, and ‘ns’ for non-significant (p ≥ 0.05).

### Supplementary Information

Supplementary Information 1.

Supplementary Video 1.

Supplementary Video 2.

Supplementary Video 3.

Supplementary Video 4.

Supplementary Video 5.

Supplementary Video 6.

Supplementary Video 7.

Supplementary Video 8.

Supplementary Video 9.

Supplementary Table 2.

Supplementary Table 3.

## Data Availability

The datasets generated during the current study are available from the corresponding author on reasonable request.
