# Supplementary material for: Neuronal substrates of egg-laying behaviour at the abdominal ganglion of Drosophila melanogaster
Source: Sci Rep. 2023 Dec 11;13:21941. doi: 10.1038/s41598-023-48109-1 (PMC10713638; doi:10.1038/s41598-023-48109-1)
Supplement: Supplementary file 1 — Supplementary Information 1. [file 41598_2023_48109_MOESM1_ESM.pdf]

Neuronal substrates of egg-laying behaviour at the abdominal ganglion of *Drosophila melanogaster*

Cristina Oliveira-Ferreira, Miguel Gaspar, Maria Luísa Vasconcelos

Supplementary Information

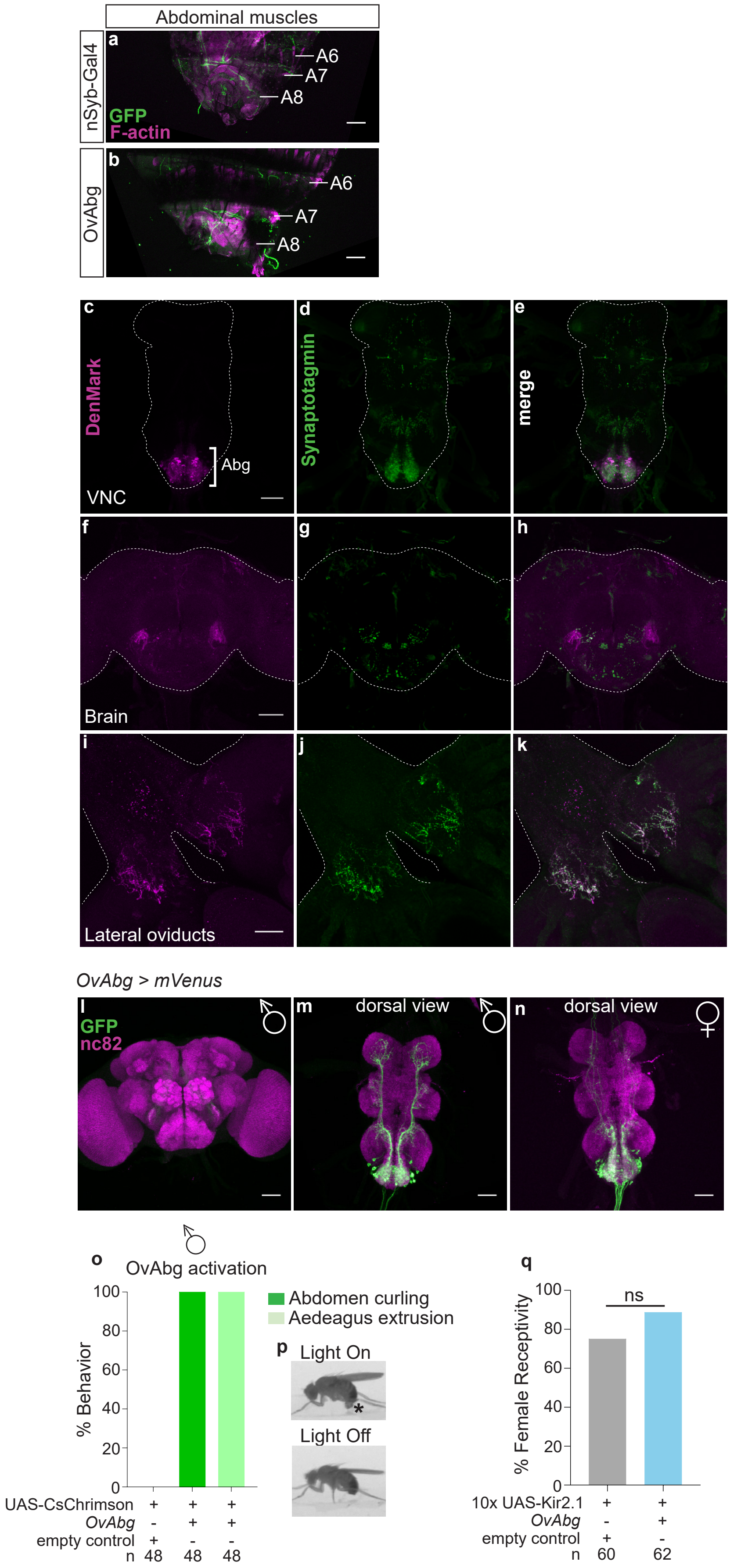

Abdominal muscles
a
nSyb-Gal4
A6
A7
A8
GFP
F-actin
b
OvAbg
A6
A7
A8
c
d
e
DenMark
Synaptotagmin
merge
Abg
VNC
f
g
h
Brain
i
j
k
Lateral oviducts
OvAbg > mVenus
l
m
n
GFP
nc82
dorsal view
dorsal view
o
OvAbg activation
% Behavior
100
80
60
40
20
0
Abdomen curling
Aedeagus extrusion
UAS-CsChrimson + + +
OvAbg - + +
empty control + - -
n 48 48 48
p
Light On
Light Off
q
% Female Receptivity
100
80
60
40
20
0
ns
10x UAS-Kir2.1 + +
OvAbg - +
empty control + -
n 60 62

Supplementary Fig. 1. Anatomical characterization of OvAbg neurons and their role in male behaviour. a and **b** Confocal images of female abdominal muscles in the Nsyb-Gal4 (**a**) and OvAbg line (**b**). Neuronal innervations are labelled with anti-GFP antibody (green) and muscle fibers with phalloidin, which binds F-actin (magenta). Abdominal segments A6, A7 and A8 are indicated. Nsyb-Gal4 line expression is shown for comparison with the OvAbg line. Anti-GFP antibody is targeting the fluorescent protein GFP and *Venus* from *Nsyb-Gal4 > UAS-mCD8::GFP* and *OvAbg > UAS-CsChrimson-mVenus* flies. Scale bars, (**a**) and (**b**) 100 µm. **c-k** Confocal images showing OvAbg neuronal polarity in the female VNC (**c-e**)**,** brain (**f-h**) and reproductive system (**i-k**). Dendrites (inputs) are labelled using the somatodendritic marker, DenMark, and axons (outputs) are labelled using the synaptic vesicle marker, Synaptotagmin. Anti-GFP and anti-DsRed antibodies are targeting EGFP-tagged Synaptotagmin and mCherry-tagged DenMark, respectively. Scale bars, (**c**), (**f**) and (**i**) 50 µm. **l-n** Confocal images of male brain (**l**) as well as male (**m**) and female (**n**) VNC (dorsal view) showing OvAbg neurons and corresponding innervations labelled with anti-GFP antibody (green) to reveal the anatomy and nc82 for neuropil. Anti-GFP antibody is targeting the fluorescent protein *Venus* from *OvAbg > UAS-CsChrimson-mVenus* expressing flies. Scale bars, (**l-n**), 50 µm. **o** Percentage of stimulation events in which male OvAbg flies displayed abdomen curling and aedeagus extrusion behaviours during photoactivation with CsChrimson. n = 48 (control) and 48 (OvAbg) stimulations. **p** Frame snapshot of an activated OvAbg male displaying abdomen curling and aedeagus extrusion (asterisk) (top). A snapshot of the same male during a light off period (below) is shown for comparison. **q** Percentage of receptive females during inhibition of OvAbg neurons. n = 60 (control) and 62 (OvAbg) females. Fisher´s exact test, ns p ≥ 0.05.

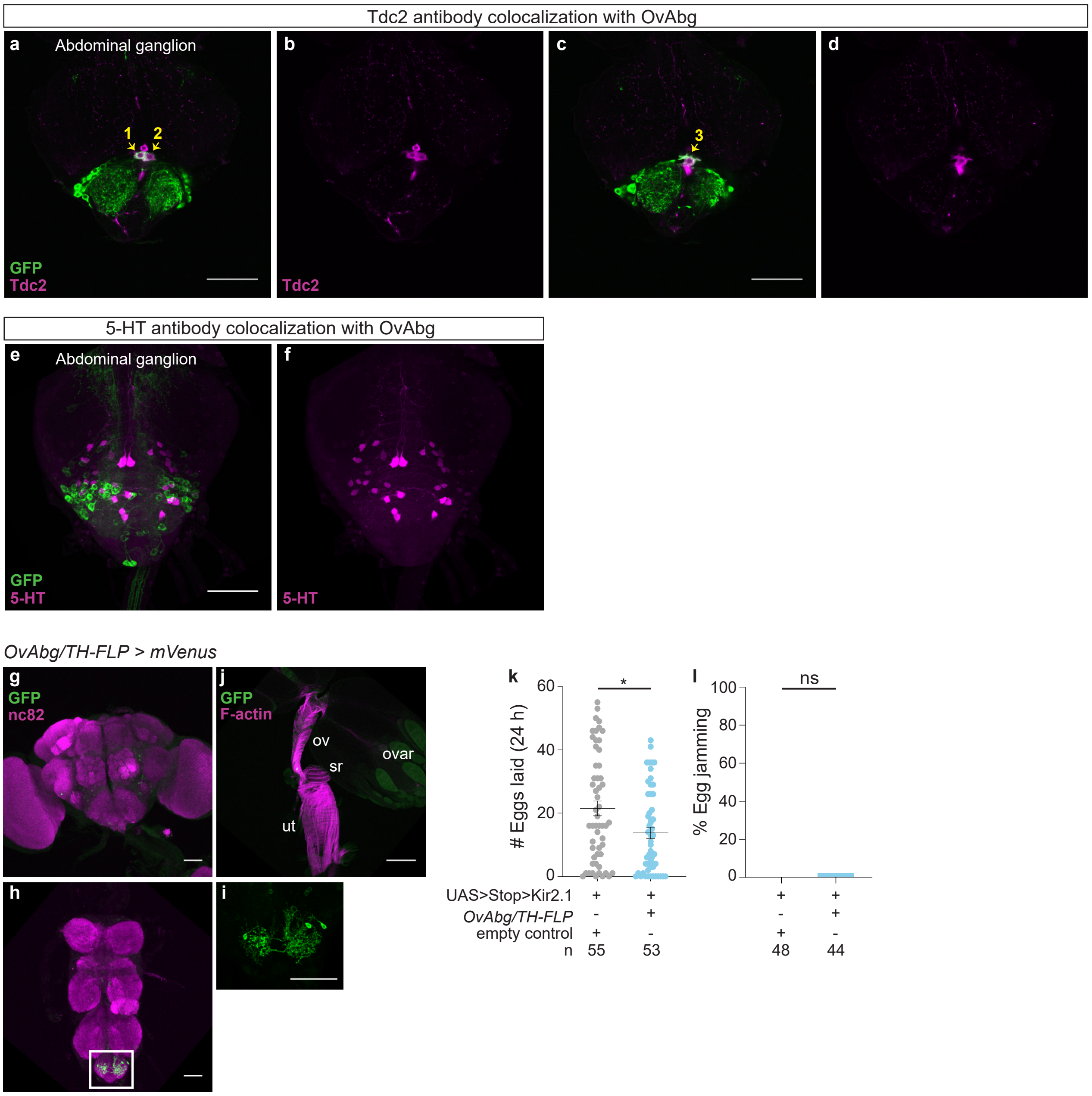
Tdc2 antibody colocalization with OvAbg
a
Abdominal ganglion
1
2
GFP
Tdc2
b
Tdc2
c
3
d
5-HT antibody colocalization with OvAbg
e
Abdominal ganglion
GFP
5-HT
f
5-HT
OvAbg/TH-FLP > mVenus
g
GFP
nc82
j
GFP
F-actin
ov
sr
ovar
ut
h
i
k
Eggs laid (24 h)
*
l
% Egg jamming
ns
UAS>Stop>Kir2.1 + + + +
OvAbg/TH-FLP - + - +
empty control + - + -
n 55 53 48 44

Supplementary Fig. 2. Neuromodulatory role of OvAbg neurons in egg-laying. a-d Colocalization of OvAbg neurons (green) with Tdc2 antibody (magenta). (**a-b**) and (**c-d**) represent different stacks of a single region of the same sample. Yellow arrows point to cells bodies of neurons colabeling for GFP and Tdc2. Scale bars, 50 μm. **e** and **f** Colocalization of OvAbg neurons (green) with 5-HT antibody (magenta). Note that there is no colabeling for GFP and 5-HT. In (**a-f**) anti-GFP antibody is targeting the fluorescent protein *Venus* from *OvAbg > UAS-CsChrimson-mVenus* expressing flies. Scale bars, 50 μm. **g-j** Anatomy of dopaminergic OvAbg neurons in the brain (**g**), VNC (**h-i**) and female reproductive system (**j**) labelled with anti-GFP antibody (green) to reveal the OvAbg anatomy and nc82 for neuropil. Phalloidin, which binds F-actin, was used in (**j**) to visualize the muscle fibres; (**i**) shows the cell bodies of dopaminergic OvAbg neurons contained in the square shown in (**h**) at higher magnification. ovar: ovary; ov: oviducts; sr: seminal receptacle; ut: uterus. Anti-GFP antibody is targeting the fluorescent protein *Venus* from *OvAbg/TH-FLP > UAS>stop>CsChrimson-mVenus* expressing flies. Scale bars, (**g-i**) 50 μm and (**j**) 200 μm. **k** Number of eggs laid in the 24h after mating during inhibition of OvAbg/TH-FLP neurons. n = 55 (control) and 53 (*OvAbg/TH-FLP*) flies. Bars indicate mean ± s.e.m.. Mann-Whitney test, *$p < 0.05$. **l** Percentage of females with eggs jammed in the lateral oviducts. n = 48 (control) and 44 (*OvAbg/TH-FLP*) flies. Fisher´s exact test, ns $p \geq 0.05$.

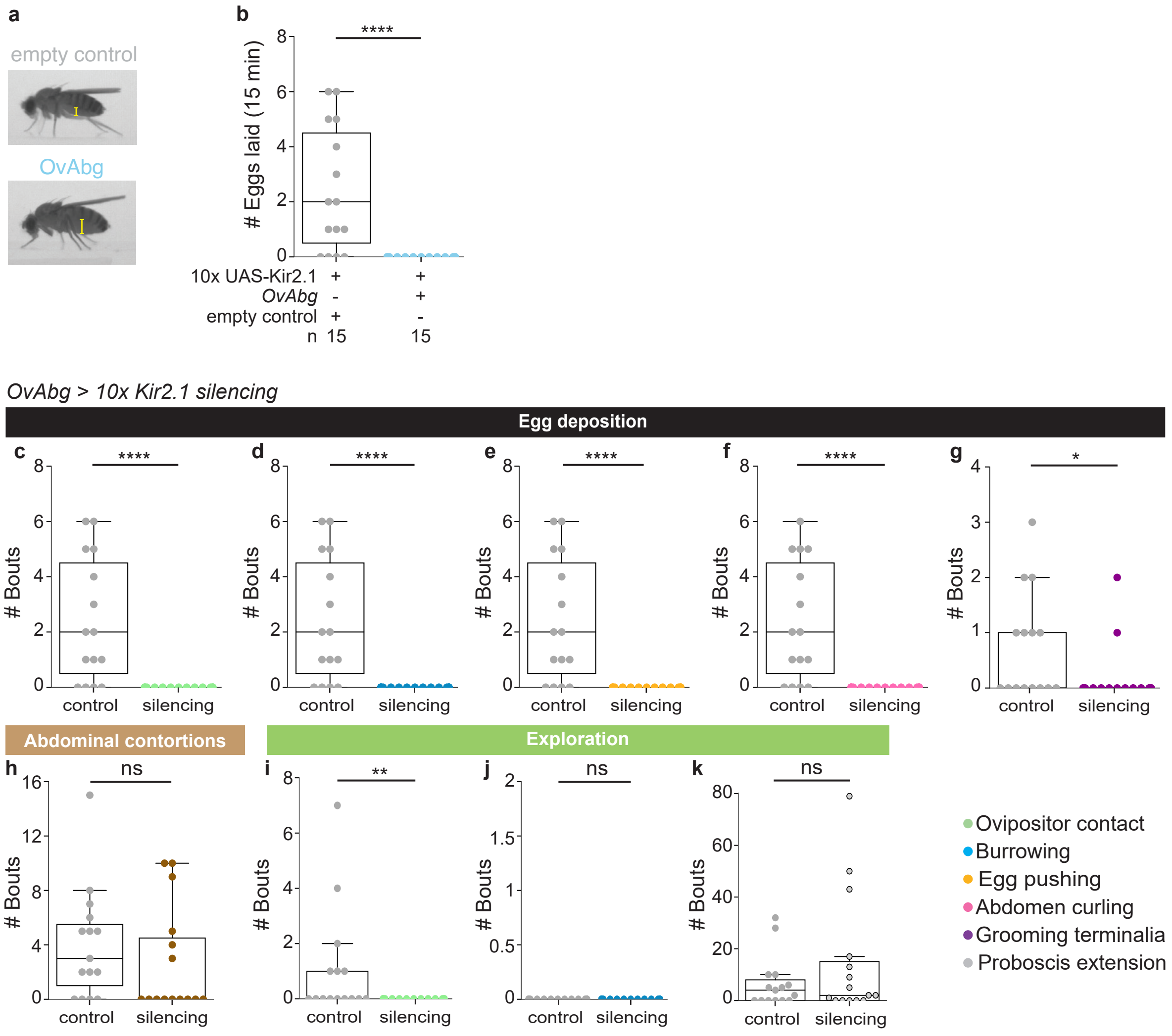

a
empty control
OvAbg
b
Eggs laid (15 min)
****
10x UAS-Kir2.1 + +
OvAbg - +
empty control + -
n 15 15
OvAbg > 10x Kir2.1 silencing
Egg deposition
c d e f g
Bouts
****
*
control
silencing
Abdominal contortions
Exploration
h i j k
ns
**
Ovipositor contact
Burrowing
Egg pushing
Abdomen curling
Grooming terminalia
Proboscis extension

Supplementary Fig. 3. Chronic silencing of OvAbg neurons disrupts several elements of the egg-laying motor programme. a Frame snapshots of control (top) and OvAbg silenced (bottom) flies. OvAbg 10xKir2.1 females display a bloated abdomen (represented by the size of the yellow bar) relative to control females. For comparison of abdomen size between control and silenced flies, the yellow bar is aligned to the A3 abdominal segment. **b** Number of eggs laid during 15 min periods. **c-g** Egg deposition phase-associated motor elements and corresponding quantification of the number of behaviour bouts. **h** Number of abdominal contortions bouts. **i-k** Exploration phase-associated motor elements and corresponding quantification of the number of behaviour bouts. The quantification of the number of behaviour bouts in (**c-k**) was quantified for the entire video duration (15 min). Box plots in (**b-k**) indicate the median (middle line), 25th, 75th percentile (box) and 5th and 95th percentile (whiskers) as well as outliers (single points). n = 15 (control) and 15 (OvAbg) flies. Unpaired t-test and Mann-Whitney test applied in normally and non-normally distributed samples, respectively. ns $p \geq 0.05$; *$p < 0.05$; **$p < 0.01$; ***$p < 0.001$; ****$p < 0.0001$ for comparisons against respective controls (grey datapoints).

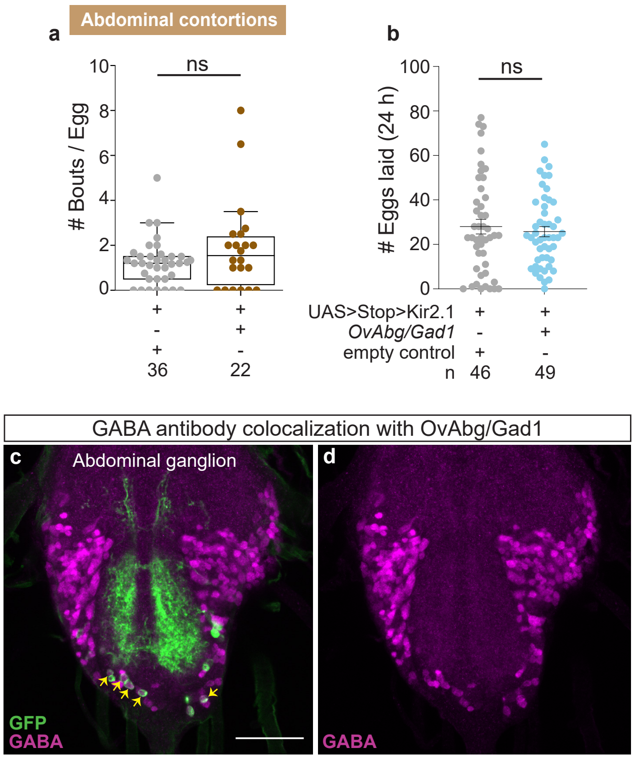

Abdominal contortions
a
b
ns
Bouts / Egg
0
2
4
6
8
10
+
-
+
36
+
+
-
22
Eggs laid (24 h)
0
20
40
60
80
100
UAS>Stop>Kir2.1 + +
OvAbg/Gad1 - +
empty control + -
n 46 49
GABA antibody colocalization with OvAbg/Gad1
c
Abdominal ganglion
GFP
GABA
d
GABA

Supplementary Fig. 4. Additional quantifications of expression and egg-laying behaviour upon silencing OvAbg/Gad1 neurons. a Number of abdominal contortions bouts during 15 min videos normalized for the number of eggs laid per female. n = 36 (control) and 22 (OvAbg/Gad1) flies. Mann-Whitney test, ns $p \geq 0.05$. Box plots indicate the median (middle line), 25th, 75th percentile (box) and 5th and 95th percentile (whiskers) as well as outliers (single points). **b** Number of eggs laid in the 24h after mating during inhibition of OvAbg/Gad1 neurons. n = 46 (control) and 49 (OvAbg/Gad1) flies. Bars indicate mean ± s.e.m.. Mann-Whitney test, ns $p \geq 0.05$. **c** and **d** Colocalization of OvAbg/Gad1 neurons (green) with GABA (magenta). A stack of a single region of the abdominal ganglion was selected and the yellow arrows in (**c**) point to cells bodies of OvAbg/Gad1 neurons co-labelling with GABA. Scale bar, 50 μm.

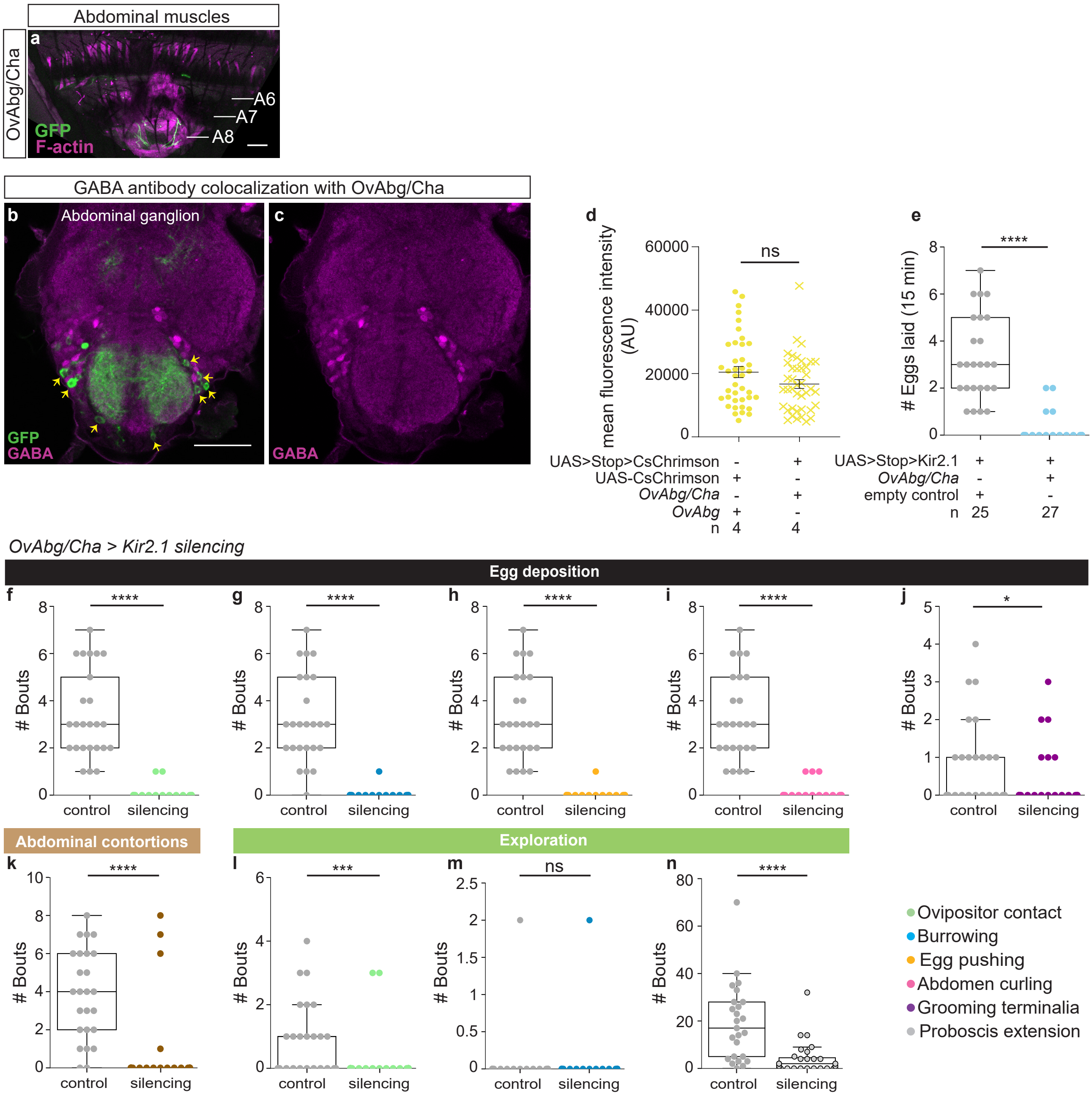

Abdominal muscles
OvAbg/Cha
a
GFP
F-actin
A6
A7
A8
GABA antibody colocalization with OvAbg/Cha
b
Abdominal ganglion
GFP
GABA
c
GABA
d
mean fluorescence intensity (AU)
60000
40000
20000
0
ns
UAS>Stop>CsChrimson - +
UAS-CsChrimson + -
OvAbg/Cha - +
OvAbg + -
n 4 4
e
Eggs laid (15 min)
****
UAS>Stop>Kir2.1 + +
OvAbg/Cha - +
empty control + -
n 25 27
OvAbg/Cha > Kir2.1 silencing
Egg deposition
f
g
h
i
j
Bouts
****
*
control
silencing
Abdominal contortions
k
Exploration
l
m
n
***
ns
Ovipositor contact
Burrowing
Egg pushing
Abdomen curling
Grooming terminalia
Proboscis extension

Supplementary Fig. 5. Chronic silencing of OvAbg/Cha neurons disrupts the egg-laying motor programme. a Confocal image of female abdominal muscles in the OvAbg/Cha line. Neuronal innervations are stained with anti-GFP antibody (green) and muscle fibers with Phalloidin, which binds F-actin (magenta). A6, A7 and A8 abdominal segments are indicated. Anti-GFP antibody is targeting the fluorescent protein *Venus* from *OvAbg/Cha* > *UAS>Stop>CsChrimson-mVenus* flies. **b** and **c** Colocalization of OvAbg/Cha neurons (green) with GABA (magenta). A stack of a single region of the abdominal ganglion was selected and the yellow arrows in (**b**) point to cells bodies of OvAbg/Cha neurons not co-labelling with GABA. **d** Mean fluorescence intensity levels of CsChrimson-mVenus expression of OvAbg and OvAbg/Cha lines measured in ten cell bodies within the same sample. n = 4 (OvAbg) and 4 (OvAbg/Cha) flies. AU: arbitrary units. Bars indicate mean ± s.e.m.. **e** Number of eggs laid during 15 min period. **f-j** Egg deposition phase-associated motor elements and corresponding quantification of the number of behaviour bouts. **k** Number of abdominal contortions bouts. **l-n** Exploration phase-associated motor elements and corresponding quantification of the number of behaviour bouts. The quantification of the number of behaviour bouts in (**f-n**) was performed for the entire video duration (15 min). Box plots in (**e-n**) indicate the median (middle line), 25th, 75th percentile (box) and 5th and 95th percentile (whiskers) as well as outliers (single points). n = 25 (control) and 27 (OvAbg/Cha) flies. Unpaired t-test and Mann-Whitney test applied in normally and non-normally distributed samples, respectively. ns P ≥ 0.05; *P < 0.05; ***P < 0.001; ****P < 0.0001 for comparisons against respective controls. Scale bars, (**a**) 100 µm; (**b-c**) 50 µm.

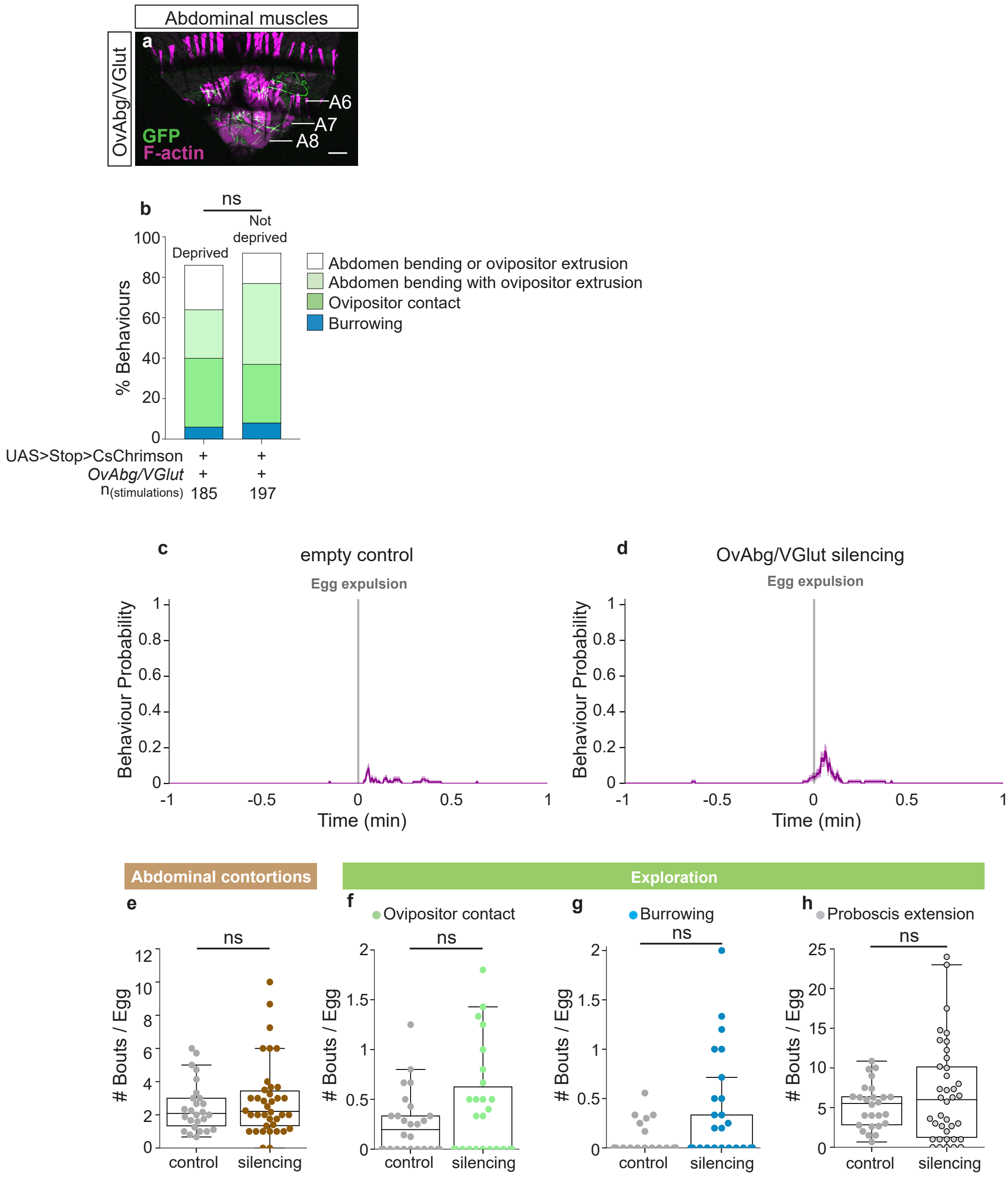

Abdominal muscles
a
OvAbg/VGlut
A6
A7
A8
GFP
F-actin
b
ns
Deprived
Not deprived
100
80
60
40
20
0
% Behaviours
Abdomen bending or ovipositor extrusion
Abdomen bending with ovipositor extrusion
Ovipositor contact
Burrowing
UAS>Stop>CsChrimson + +
OvAbg/VGlut + +
n(stimulations) 185 197
c
empty control
Egg expulsion
Behaviour Probability
Time (min)
d
OvAbg/VGlut silencing
Egg expulsion
Behaviour Probability
Time (min)
Abdominal contortions
Exploration
e
ns
Bouts / Egg
control
silencing
f
Ovipositor contact
ns
Bouts / Egg
control
silencing
g
Burrowing
ns
Bouts / Egg
control
silencing
h
Proboscis extension
ns
Bouts / Egg
control
silencing

Supplementary Fig. 6. Effect of manipulating OvAbg/VGlut activity on egg-laying behaviour. a Confocal image of female abdominal muscles in the OvAbg/VGlut line. Neuronal innervations are stained with anti-GFP antibody (green) and muscle fibres with Phalloidin, which binds F-actin, (magenta). A6, A7 and A8 abdominal segments are indicated. Anti-GFP antibody is targeting the fluorescent protein *Venus* from *OvAbg/VGlut* > *UAS>Stop>CsChrimson-mVenus* flies. Scale bar, 100 µm. **b** Percentage of stimulation periods in which egg-laying deprived and not deprived OvAbg/VGlut females displayed ovipositor contact, burrowing, abdomen bending with ovipositor extrusion and abdomen bending or ovipositor extrusion behaviours. n = 185 (deprived), 197 (not deprived) stimulations. Fisher´s exact test, ns $p \geq 0.05$. **c-d** Probabilities of grooming terminalia behaviour during a 1-min time window around egg expulsion for (**c**) control and (**d**) OvAbg/VGlut silenced flies. Time = 0 min marks the moment of egg expulsion (represented by the grey vertical line). Shaded area represents the standard error of the mean (s.e.m.). n = 126 (control) and 105 (OvAbg/VGlut) egg expulsions. **e** Number of abdominal contortions bouts. **f-h** Exploration phase-associated motor elements and corresponding quantification of the number of behaviour bouts. In (**e-h**) the quantification of the number of behaviour bouts was normalized for the number of eggs laid per female and quantified for the entire video duration (15 min). n = 26 (control) and 38 (OvAbg/VGlut) females. Box plots indicate the median (middle line), 25th, 75th percentile (box) and 5th and 95th percentile (whiskers) as well as outliers (single points). Mann-Whitney test, ns $p \geq 0.05$ for comparisons against respective controls (grey datapoints).

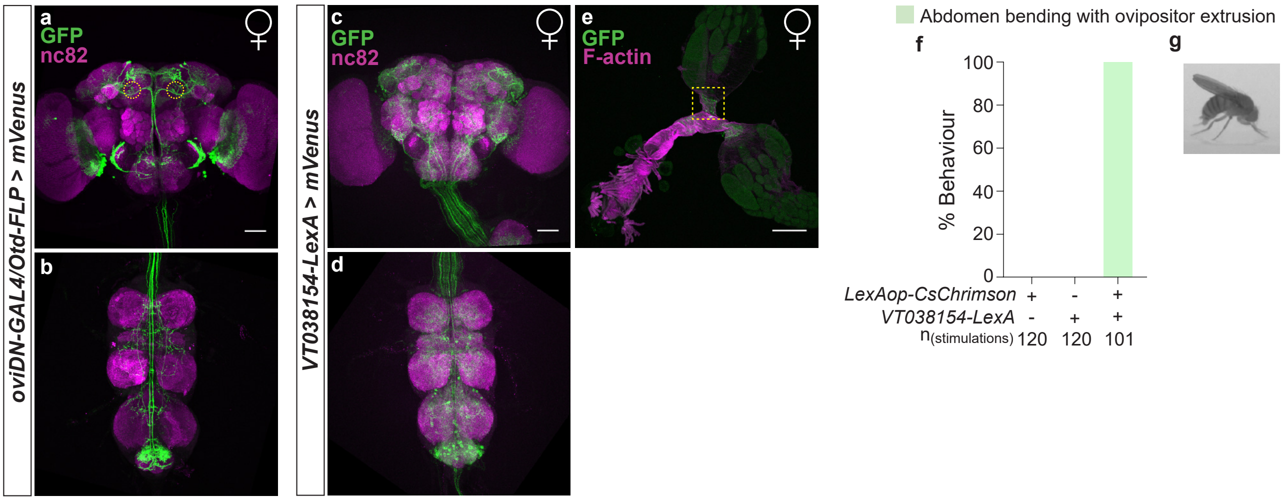

oviDN-GAL4/Otd-FLP > mVenus
VT038154-LexA > mVenus
a
GFP
nc82
b
c
GFP
nc82
d
e
GFP
F-actin
Abdomen bending with ovipositor extrusion
f
g
% Behaviour
100
80
60
40
20
0
LexAop-CsChrimson + - +
VT038154-LexA - + +
n(stimulations) 120 120 101

Supplementary Fig. 7. Anatomy and function of lines used in figure 7. a-b Confocal images of oviDN-Gal4/Otd-FLP anatomy in the female brain (**a**) and VNC (**b**). Yellow circles indicate oviDNs' cell body location in the brain. Genotype: *oviDN-Gal4/Otd-FLP > UAS>Stop>CsChrimson-mVenus* flies. **c-e** VT038154-LexA anatomy in the female brain (**c**), VNC (**d**) and reproductive system (**e**). Yellow box in (**e**) indicates the presence of innervations in the lateral oviducts. Genotype: *VT038154-LexA > LexAop-CsChrimson-mVenus* flies. In (**a-e**) neuronal innervations were stained with anti-GFP antibody (green) targeting the fluorescent protein *Venus* and nc82 for neuropil. Phalloidin, which binds F-actin, was used in the reproductive system to visualize the muscle fibres. Scale bars, (**a-d**), 50 μm; **e**, 200 μm. **f** Percentage of stimulation events in which VT038154-LexA activated females displayed abdomen bending with ovipositor extrusion (not touching the substrate). The controls are each parental line crossed with *white (w-)* background. n = 120 (LexAop-CsChrimson-mVenus control), 120 (VT038154-LexA control) and 101 (test) stimulations. **g** Frame snapshot of an activated VT038154-LexA female displaying abdomen bending with ovipositor extrusion not touching the substrate. Note that VT038154-LexA activated females only performed this behaviour in response to the light stimulation.

Supplementary Table 1 | Fly stocks

Stocks	Source	Reference
Canton S (CS)	Lab stock	-
w-; empty-p65-AD(attp40); +	BDSC, 71210	Hampel et al[1]
w-; VT038154-p65.AD(attp40); +	BDSC, 74056	Tirian et al[2]
w-; VT038154-LexA(attp40); +	From this study	-
w-;+; dsx^{DBD}	Provided by Stephen Goodwin	Pavlou et al[3]
w-;VT040574-GAL4(attp2) (oviDN-GAL4)	Provided by Gaby Maimon	Vijayan et al[4]<br>Tirian et al[2]
w-; +; ppk-LexA	Provided by Jay Parrish	Yoshino et al[5]
w-;TH-FLP; +	BDSC, 93706	Xie et al[6]
y^{1}*w-; +; nSyb-GAL4*	BDSC, 51941	-
w-; Otd-nls:FLPo(attp40); +	Provided by David Anderson	Asahina et al[7]
y^{1}*w-; Gad1(MI09277)-LexA:QFAD; +*	BDSC, 60324	Diao et al[8]
y^{1}*w-; ChaT(MI04508)-LexA:QFAD; +*	BDSC, 60319	Diao et al[8]
y^{1}*w-; VGlut(MI04979)-LexA:QFAD; +*	BDSC, 60314	Diao et al[8]
w-;; 20XUAS-IVS-CsChrimson-mVenus(attp2)	BDSC, 55136	Klapoetke et al[9]
w-, 20XUAS-IVS-CsChrimson-mVenus(attP18);+;+	BDSC, 55134	Markstein et al[10]
w-;; 20XUAS-FRT-stop-FRT-CsChrimson-mVenus(attp2)	FLP-out version provided by Vivek Jayaraman	Klapoetke et al[9]
w-;;13XLexAop2-IVS-CsChrimson-mVenus(attp2)	BDSC, 55139	-
w-;13XLexAop2-IVS-CsChrimson-mVenus(attp40); +	BDSC, 55138	-
DL; +; 10XUAS-IVS-eGFPKir2.1	Provided by Marta Moita	von Reyn et al[11]
w-;UAS>Stop>Kir2.1-EGFP(VIE-19A);+	Provided by Eugenia Chiappe	Asahina et al[7]
w-; +; 20xUAS-GtACR1(attp2)	Provided by Carlos Ribeiro	Mohammad et al[12]
w-; 8xLexAop2-FLP(attp40); +	BDSC, 55820	Pan et al[13]

w-; +; 8xLexAop2-FLP(attp2)	BDSC, 55819	Pan et al[13]
w-; UAS-mCD8::GFP; +	BDSC, 5137	Lee et al[14]
w-; UAS-DenMark2, UAS-syt.EGFP	BDSC, 33064	Nicolai et al[15]
w-;; UAS-CD4::spGFP1-10	BDSC, 93016	Gordon et al[16]
w-; UAS-CD4::spGFP1-10; +	BDSC, 93017	Gordon et al[16]
w-; LexAop-CD4::SpGFP11; +	BDSC, 93019	Gordon et al[16]
w-;; LexAop-CD4::SpGFP11	BDSC, 93018	Gordon et al[16]
y[1] w[*]; wg[Sp-1]/CyO; 20XUAS-post-t-GRASP}attP2 13XLexAop2-pre-t-GRASP}VK00027/TM6C, Sb[1] Tb[1]	BDSC, 79039	Shearin et al[17]
y[1] w[*]; wg[Sp-1]/CyO; 13XLexAop2-post-t-GRASP}attP2 20XUAS-pre-t-GRASP}VK00027	BDSC, 79040	Shearin et al[17]

BDSC - Bloomington Drosophila Stock Center

1. Hampel, S., Franconville, R., Simpson, J. H. & Seeds, A. M. A neural command circuit for grooming movement control. *eLife* **4**, e08758 (2015).
2. Tirian, L. & Dickson, B. J. The VT GAL4, LexA, and split-GAL4 driver line collections for targeted expression in the Drosophila nervous system. *bioRxiv* (2017) doi:10.1101/198648.
3. Pavlou, H. J. *et al.* Neural circuitry coordinating male copulation. *eLife* **5**, e20713 (2016).
4. Vijayan, V. *et al.* A rise-to-threshold signal for a relative value deliberation. *Nature* **619**, 563-571 (2023).
5. Yoshino, J., Morikawa, R. K., Hasegawa, E. & Emoto, K. Neural Circuitry that Evokes Escape Behavior upon Activation of Nociceptive Sensory Neurons in Drosophila Larvae. *Curr. Biol.* **27**, 2499-2504.e3 (2017).
6. Xie, T. *et al.* A Genetic Toolkit for Dissecting Dopamine Circuit Function in Drosophila. *Cell Rep.* **23**, 652–665 (2018).
7. Asahina, K. *et al.* Tachykinin-Expressing Neurons Control Male-Specific Aggressive Arousal in Drosophila. *Cell* **156**, 221–235 (2014).
8. Diao, F. *et al.* Plug-and-Play Genetic Access to Drosophila Cell Types using Exchangeable Exon Cassettes. *Cell Rep.* **10**, 1410–1421 (2015).
9. Klapoetke, N. C. *et al.* Independent optical excitation of distinct neural populations. *Nat. Methods* **11**, 338–346 (2014).
10. Markstein, M., Pitsouli, C., Villalta, C., Celniker, S. E. & Perrimon, N. Exploiting position effects and the gypsy retrovirus insulator to engineer precisely expressed transgenes. *Nat. Genet.* **40**, 476–483 (2008).
11. von Reyn, C. R. *et al.* A spike-timing mechanism for action selection. *Nat. Neurosci.* **17**, 962–970 (2014).

12. Mohammad, F. *et al.* Optogenetic inhibition of behavior with anion channelrhodopsins. *Nat. Methods* **14**, 271–274 (2017).

13. Pan, Y., Meissner, G. W. & Baker, B. S. Joint control of Drosophila male courtship behavior by motion cues and activation of male-specific P1 neurons. *Proc. Natl. Acad. Sci.* **109**, 10065–10070 (2012).

14. Lee, T. & Luo, L. Mosaic Analysis with a Repressible Cell Marker for Studies of Gene Function in Neuronal Morphogenesis. *Neuron* **22**, 451–461 (1999).

15. Nicolai, L. J. J. *et al.* Genetically encoded dendritic marker sheds light on neuronal connectivity in Drosophila. *Proc. Natl. Acad. Sci.* **107**, 20553–20558 (2010).

16. Gordon, M. D. & Scott, K. Motor Control in a Drosophila Taste Circuit. *Neuron* **61**, 373–384 (2009).

17. Shearin, H. K. *et al.* t-GRASP, a targeted GRASP for assessing neuronal connectivity. Journal of Neuroscience Methods **306**, 94-102 (2018).
